# p38 MAPK pathway and its interaction with TRF2 in cisplatin induced chemotherapeutic response in head and neck cancer

**DOI:** 10.1038/s41389-018-0062-6

**Published:** 2018-07-09

**Authors:** Shomereeta Roy, Souvick Roy, Madhabananda Kar, Shweta Thakur, Yusuf Akhter, Amit Kumar, Francesco Delogu, Swatishree Padhi, Arka Saha, Birendranath Banerjee

**Affiliations:** 1 Molecular Stress and Stem Cell Biology Group, School of Biotechnology, KIIT University, Bhubaneswar, Odisha 751024 India; 2 Professor and Head, Department of Surgical Oncology, All India Institute of Medical Sciences (AIIMS), Bhubaneswar, Odisha 751019 India; 3 Centre for Computational Biology and Bioinformatics, School of Life Sciences, Central University of Himachal Pradesh, Shahpur, Himachal Pradesh 176206 India; 4 Department of Biotechnology, Babasaheb Bhimrao Ambedkar University, Vidya Vihar, Raebareli Road, Lucknow, Uttar Pradesh 226025 India; 5 Departments of Mechanical, Chemical and Materials Engineering, University of Cagliari, via Marengo 2, 09123 Cagliari, Italy; 6 Biosciences Sector, Center for Advanced Study Research and Development in Sardinia (CRS4), Loc. Piscina Manna, 09010 Pula, Italy

## Abstract

TRF2 is a telomere binding protein, a component of the shelterin complex that plays a major role in maintaining the integrity of the genome. TRF2 is over-expressed in a number of human cancers including Head and Neck cancer and might play a key role in tumor initiation and development. p38 MAPK signaling pathway is strongly activated in response to various environmental and cellular stresses and thus overexpressed in most of the Head and Neck cancer cases. In this study, we investigated potential interactions of TRF2 with p38 in HNSCC cells and patient samples. Using in silico experiments, we identified interface polar residue Asp-354 of p38 and Arg-492, Arg-496 of TRF2 as protein–protein interaction hotspots. In addition to these interactions, Arg-49 residue of p38 was also found to interact with Glu-456 of TRF2. A detailed understanding of how phosphorylated and unphosphorylated state of p38 protein can influence the stability, specificity and to some extent a conformational change of p38-TRF2 binding is presented. Silencing of TRF2 significantly decreased the phosphorylation of p38 in HNSCC cells which was confirmed by western blot, immunofluorescence and co-immunoprecipitation and alternatively inhibiting p38 using p38 inhibitor (SB 203580) decreased the expression of TRF2 in HNSCC cells. Furthermore, we checked the effect of TRF2 silencing and p38 inhibition in cisplatin induced chemosensitivity of SCC-131 cells. TRF2 silencing and p38 inhibition chemosensitize HNSCC cells to cisplatin. Thus, targeting TRF2 in combinatorial therapeutics can be a treatment modality for Head and Neck cancer which involves inhibition of p38 MAPK pathway.

## Introduction

Head and neck squamous cell carcinoma (HNSCC) is the sixth most prevalent cancer in the world^1,2^. Despite advancements in treatment modalities, prognosis remains poor due to recurrence and invasion^3^. India has a higher rate of HNSCC due to the habits of tobacco chewing and smoking^1^. Continuous smoking and exposure to tobacco induces oxidative stress causing DNA damage, activation of MAPK pathway and dysfunctional telomere thereby playing an intricate role in carcinogenesis^4,5^.

In response to DNA damage telomere plays a crucial to maintain chromosomal integrity and is protected by shelterin complex^6,7^. Telomere Repeat Binding Factor 2 (TRF2), a component of shelterin complex, interacts with distal end of chromosome and prevents the telomeres from being recognized as a double-strand break^8^. In normal cells, loss of TRF2 function leads to activation of an array of DNA repair machinery specifically at telomeric loci, leading to cell cycle arrest, senescence and cell death^9,10^. TRF2 over-expression was observed in different human cancers like lung cancer and gastric cancer suggesting a crucial role of TRF2 in tumor initiation and development^11,12^. In a previous study it has been reported that inhibition of TRF2 expression reduced cell proliferation and migration and induced apoptosis in renal cell carcinoma^13^.

In accordance with the evidence that 80% of HNSCCs are also associated with over-expression and activation of the several signaling pathways such as mitogen-activated protein kinase (MAPK), epidermal growth factor receptor (EGFR), and PI3 Kinase/AKT signaling pathways^14^. A key member of MAPK family, p38 is strongly activated in response to various environmental and cellular stresses, inflammation, and other signals^15^. Activation of p38 MAPK has been reported to be essential for survival of cells in response to DNA damage^16^. DNA damage causes phosphorylation of p38 MAPK and its nuclear translocation^17^. p38 MAPK was found to be activated in most HNSCC cases and the blockage of p38 signaling was noted to significantly inhibit the proliferation of cancer cells both in vitro and in vivo^2^. Earlier studies have reported a significant role of p38 in modulating expression levels of TRF2^18–20^. In a recent study, it has been observed that mice subjected to physiological stressors exhibited an increased levels of TRF1 and TRF2 proteins, and of mRNA levels along with a greater protein content of phosphorylated p38^21^. In addition, an important role of TRF2 is familiar in the DNA damage response of tumors^22^ which is also influenced by p38 MAPK pathway as stress response to DNA damaging agents. Therefore, it is important to study the interactive and regulatory roles if any between these two molecules.

In this study, we investigated the interaction between telomeric TRF2 and the stress molecule p38 in HNSCC. We observed interactions between p38 and TRF2 molecules in HNSCC cell line and in HNSCC patient samples. To provide an atomistic level description of p38–TRF2 interaction, we utilized molecular docking and molecular dynamics (MD) simulations on protein- protein complexes, which confirmed the potential interactions between these proteins. Furthermore, we analysed the binding affinity, stability differences and conformational changes upon interaction of TRF2 protein with phosphorylated and unphosphorylated forms of p38 MAPK. In addition, to validate the role of TRF2 and p38 in chemosensitivity or drug response, we investigated the effect of cisplatin in HNSCC cell line for head and neck cancer treatment^23^.

## Results

### p-p38 and TRF2 interact with each other in HNSCC cell lines

**Fig. 1:**
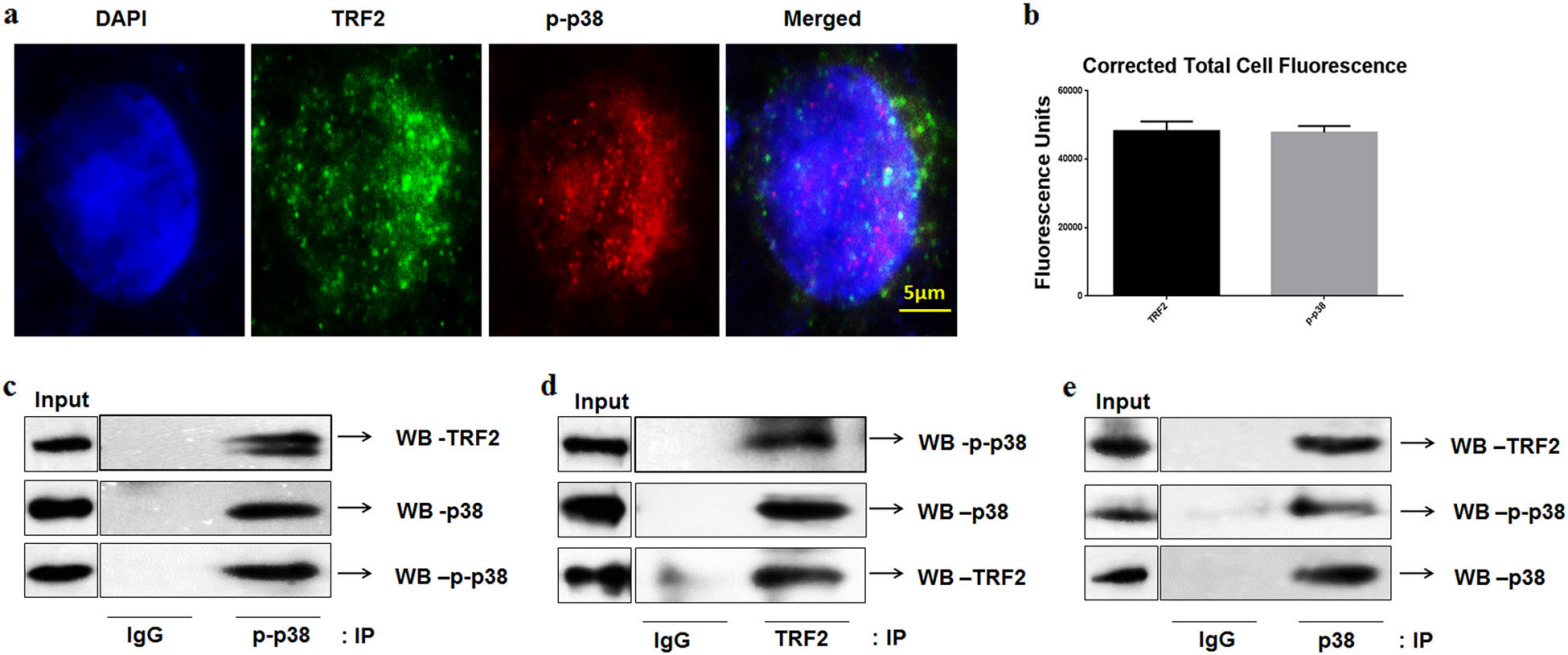
TRF2 and p-p38 co-localizes and co-immunoprecipitates in SCC-131 cells. **a** Representative immunofluorescence images of SCC-131 cells co-stained for TRF2 and p-p38. Merged images indicate co-localization. DAPI was used to visualize nuclear DNA. **b** Corrected total cell fluorescence showing the expression of TRF2 and p-p38 in SCC-131 cells. **c** p-p38 was immunoprecipitated from SCC-131 cell’s protein extract and was immunoblotted with TRF2, p-p38, and p38 antibody. **d** TRF2 was immunoprecipitated from SCC-131 cell’s protein extract and was immunoblotted with p-p38, p38, and TRF2 antibody. **e** p38 was immunoprecipitated from SCC-131 cell’s protein extract and was immunoblotted with p-p38, TRF2, and p38 antibody

In this study, different strategies were employed to visualize protein-protein interactions between p38 and TRF2. The activated form of p38 (p-p38) was found to co-localize with TRF2 in the nucleus of SCC-131 cells and CAL 27 cells when immunostained with p-p38 (Thr180/Tyr182) and TRF2 antibody respectively (Fig. 1a, b, Supplementary Figure S1a and b). The co-localization of p-p38 and TRF2 suggested that activated form of p38 might interact with TRF2 which was further confirmed by co-immunoprecipitation (Co-IP) assay in SCC-131 cells. The immunoprecipitated lysates were immunoblotted with p-p38, p38, and TRF2 (Fig. 1c–e). The results obtained from Co-IP demonstrated an interaction of TRF2 with p-p38 in HNSCC cells.

### Silencing of TRF2 affect phosphorylation of p38 in HNSCC cell lines

**Fig. 2:**
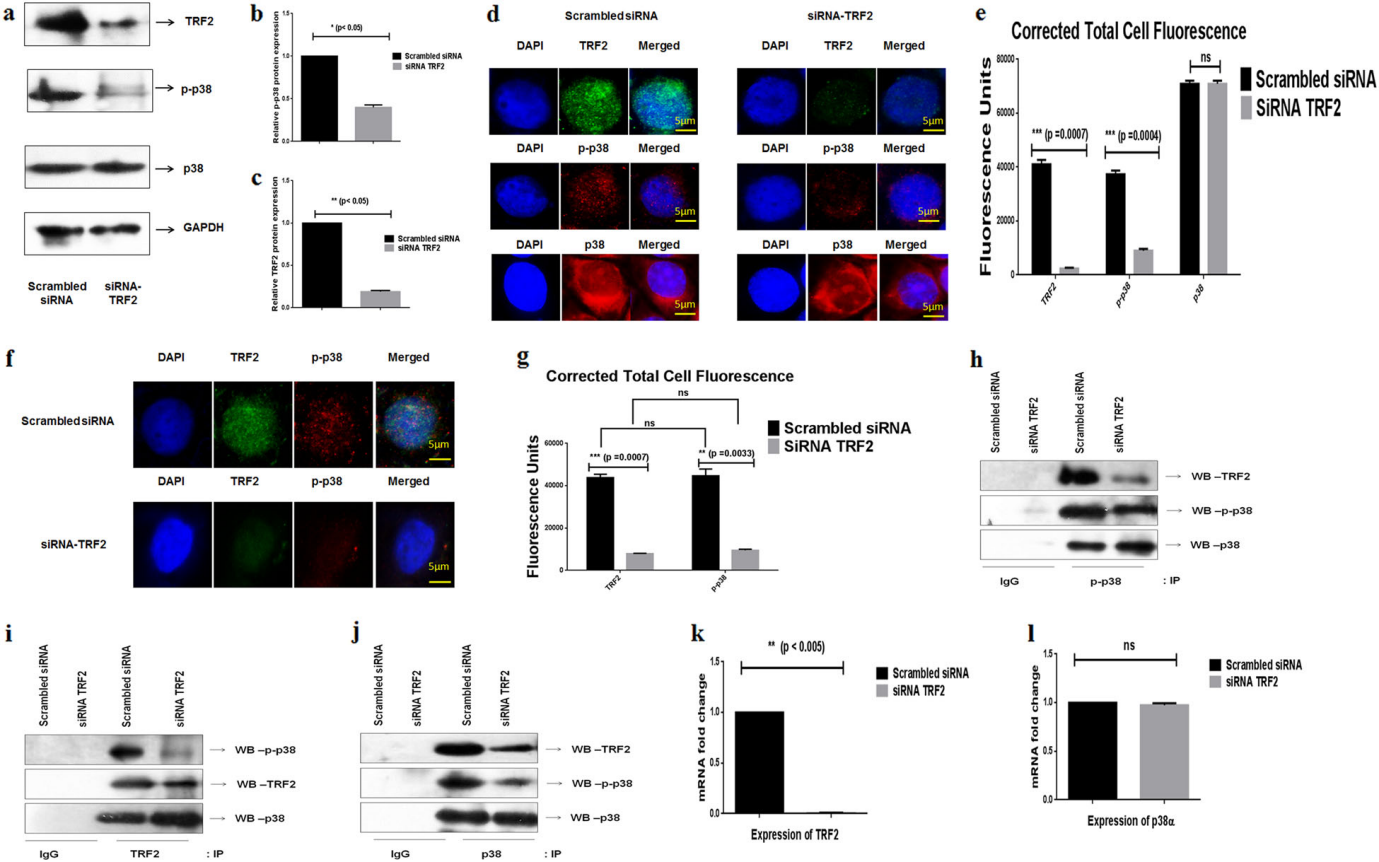
TRF2 silencing decreases phosphorylation of p38 in SCC-131 cells. **a** Representative blots showing decreased expression of TRF2 and p38 phosphorylation in whole cell extracts after silencing TRF2. **b**, **c** Relative TRF2 expression and p38 phosphorylation in whole cell extracts post TRF2 silencing. **d** Representative immunofluorescence images of cells showing decreased expression of TRF2 and p38 phosphorylation after TRF2 silencing. **e** Corrected total cell fluorescence showing significantly decreased expression of TRF2 and p38 phosphorylation in TRF2 silenced SCC-131 cells. **f** Representative immunofluorescence images of cells showing decreased co-localization of TRF2 and p-p38 in TRF2 silenced cells. **g** Corrected total cell fluorescence showing decreased co-localization of TRF2 and p-p38 in TRF2 silenced cells. **h** p-p38 was immunoprecipitated from protein extract of cells transfected with scrambled siRNA and siRNA TRF2 and was immunoblotted with TRF2, p-p38, and p38. **i** TRF2 was immunoprecipitated from protein extract of cells transfected with scrambled siRNA and siRNA TRF2 and was immunoblotted with p-p38, p38, and TRF2. **j** p38 was immunoprecipitated from protein extract of cells transfected with scrambled siRNA and siRNA TRF2 and was immunoblotted with TRF2, p-p38, and p38. Co-immunoprecipitation revealed interaction between TRF2 and p-p38. **k** Quantitative Real Time PCR analysis showing decreased expression of TRF2 after TRF2 silencing. **l** Quantitative Real time PCR analysis showing no effect in the expression p38α gene after TRF2 silencing. *Y*-axis represents fold change in mRNA expression. Data presented is the mean ± SD of three independent experiments. Statistical significance was determined by two way ANOVA test (*p *<* 0.05), (**p *<* 0.005), (*** p *<* 0.001)

To assess whether depletion of TRF2 would affect phosphorylation of p38 MAPK, HNSCC cells were transiently silenced with TRF2 siRNA. Transfection efficiency was checked by Western Blot (WB) analysis (Fig. 2a, c, Supplementary Figure S2a and c), immunofluorescence assay (Fig. 2d and Supplementary Figure S2d) and quantitative Real Time PCR (Fig. 2k, Supplementary Figure S2h) after 48 h. Phosphorylation of p38 was observed to be decreased in siTRF2 transfected HNSCC cells as compared to the scrambled counterpart by WB analysis (Fig. 2a, b, Supplementary Figure S2a and b). Immunofluorescence assay also showed significant reduction in phosphorylation of p38 and TRF2 expression along with decreased co-localization of TRF2 and p-p38 in siTRF2 transfected cells as compared to the scrambled counterpart (Fig. 2d–g, Supplementary Figure S2d–g).To bolster our findings, we further performed Co-IP assays in SCC-131 cells. Co-IP post siTRF2 silencing resulted in loss of p38 phosphorylation post pull down with TRF2 antibody and vice versa (Fig. 2h, i). The expression of TRF2 and p38 phosphorylation was also reduced in siTRF2 silenced SCC-131 cells post pull down with total p38 antibody (Fig. 2j). These observations indicated that TRF2 indeed interacts with p-p38 and silencing of TRF2 diminishes the phosphorylation of p38 in HNSCC cells.

Gene expression study showed no significant change in the expression of p38α gene in siTRF2 transfected HNSCC cells as compared to the scrambled counterpart (Fig. 2l and Supplementary Figure S2i). Thus, suggesting that TRF2 silencing effects the phosphorylation of p38 and had no effect on the expression of total p38.

### Inhibition of p38, affect TRF2 expression in HNSCC cells

**Fig. 3:**
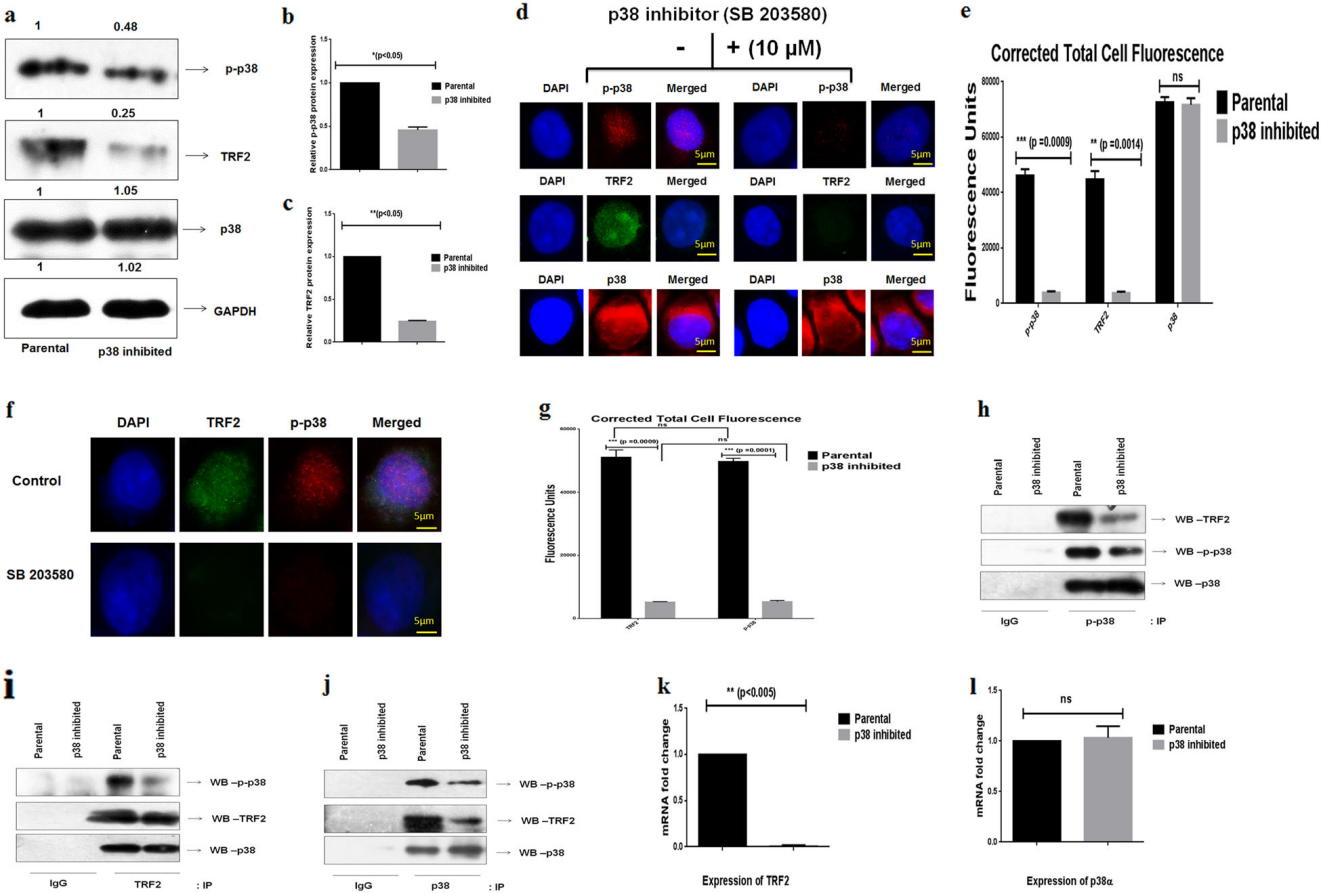
p38 inhibition decreases expression of TRF2 in SCC-131 cells. **a** Representative blots showing decreased p38 phosphorylation and TRF2 in whole cell extracts after inhibiting p38. **b**, **c** Relative p38 phosphorylation and TRF2 expression in whole cell extracts after p38 inhibition. **d** Representative immunofluorescence images of cells showing decreased expression of TRF2 and p38 phosphorylation after p38 inhibition. **e** Corrected total cell fluorescence showing significantly decreased p38 phosphorylation and TRF2 expression in p38 inhibited SCC-131 cells. **f** Representative immunofluorescence images of cells showing decreased co-localization of TRF2 and p-p38 in p38 inhibited cells. **g** Corrected total cell fluorescence showing decreased co-localization of TRF2 and p-p38 in p38 inhibited cells. **h** p-p38 was immunoprecipitated from protein extract of parental SCC-131 cells and p38 inhibited SCC-131 cells and was immunoblotted with TRF2, p-p38, and p38 antibody. **i** TRF2 was immunoprecipitated from protein extract of parental SCC-131 cells and p38 inhibited SCC-131 cells and was immunoblotted with p-p38, TRF2, and p38 antibody. **j** p38 was immunoprecipitated from protein extract of parental SCC-131 cells and p38 inhibited SCC-131 cells and was immunoblotted with p-p38, TRF2, and p38 antibody. Co-immunoprecipitation studies in parental SCC-131 and p38 inhibited SCC-131 cells revealed interaction between p-p38 and TRF2. **k** Quantitative real time PCR analysis showing decreased expression of TRF2 gene after p38 inhibition. **l** Quantitative real time PCR analysis showing no effect in the expression p38α gene after p38 inhibition. *Y*-axis represents fold change in mRNA expression. Data presented is the mean ± SD of three independent experiments. Statistical significance was determined by two way ANOVA test (*p *<* 0.05), (**p *<* 0.005), (*** p *<* 0.001)

Alternatively, to check whether p38 inhibition has any affect in TRF2 expression, p38 was inhibited in SCC-131 cells. The inhibition was confirmed by WB analysis (Fig. 3a, b) and immunofluorescence assay (Fig. 3d). Significant downregulation of TRF2 protein expression was observed in p38 inhibited HNSCC cells, as compared to parental cells (Fig. 3a–e) along with reduced co-localization of TRF2 and p-p38 (Fig. 3f, g). Further, Co-IP post p38 inhibition resulted in loss of TRF2 expression in p38 inhibited SCC-131 cells post pull down with p-p38 antibody and vice versa (Fig. 3h, i). The expression of TRF2 and p38 phosphorylation was also less in p38 inhibited SCC-131 cells as compared to parental cells post pull down with total p38 antibody (Fig. 3j). Gene expression studies also showed downregulation of TRF2 gene in p38 inhibited cells as compared to parental cells (Fig. 3k) but no effect was observed on the expression of p38α gene (Fig. 3l).

### Interaction of TRF2 with p38/MAPK in human head and neck squamous cell carcinoma (HNSCC) patient samples

**Fig. 4:**
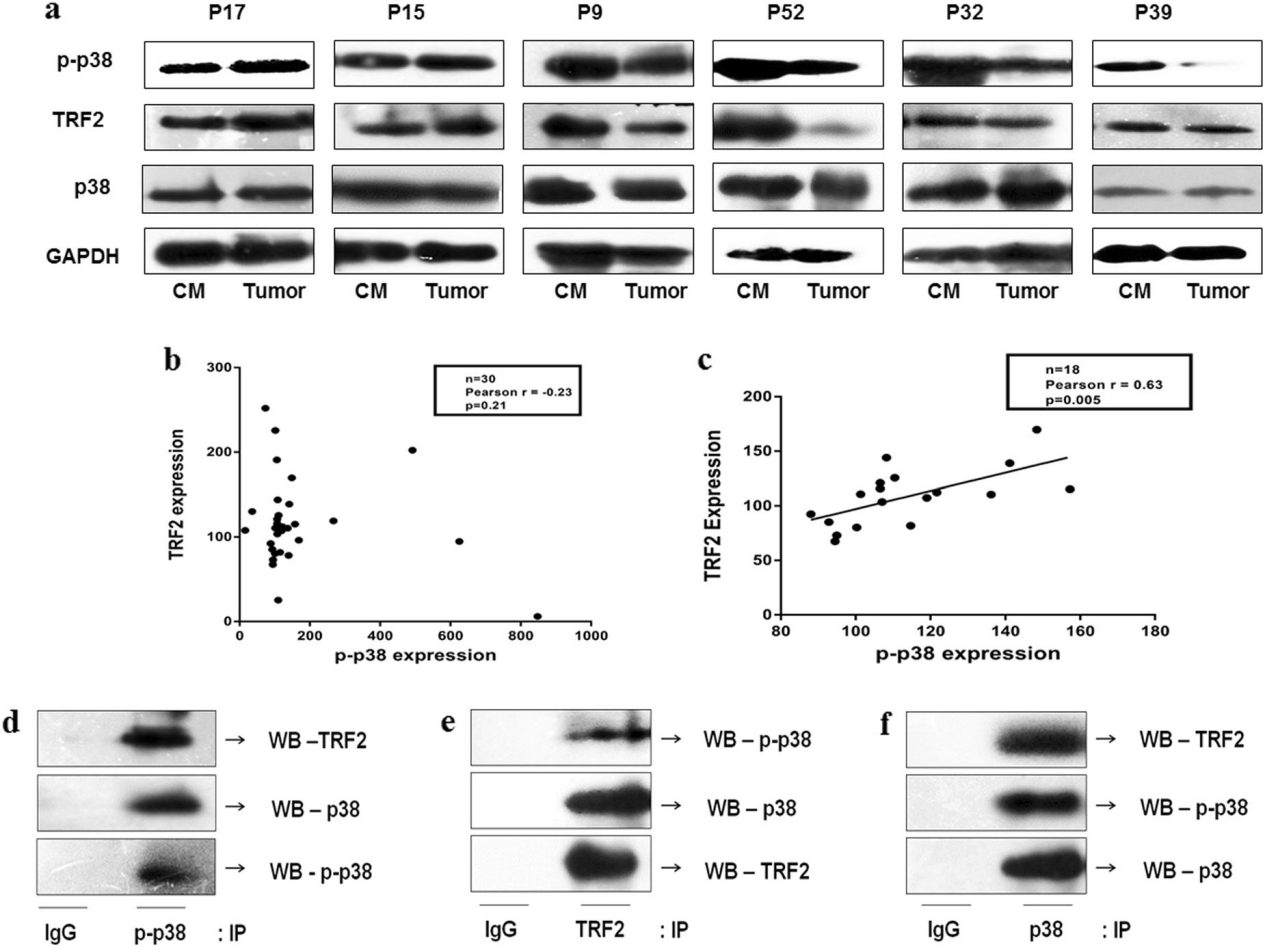
Correlation and interaction analysis of TRF2 expression and phosphorylated p38 in HNSCC patient samples. **a** Representative blots showing the p38 phosphorylation and TRF2 in HNSCC samples for their respective tumor and cut margin counterparts. **b** Scatterplot of the expression of TRF2 vs p38 phosphorylation in 30 HNSCC samples. **c** Eighteen cases showing a positive correlation for the expression of TRF2 and p38 phosphorylation (*p* = 0.005). **d** p-p38 was immunoprecipitated from tumor extract and was immunoblotted with TRF2, p38, and p-p38 antibody. **e** TRF2 was immunoprecipitated from tumor extract and was immunoblotted with p-p38, p38, and TRF2 antibody. **f** p38 was immunoprecipitated from tumor extract and was immunoblotted with TRF2, p-p38, and p38 antibody. The co-immunoprecipitation studies confirmed the interaction of TRF2 with p-p38

The observations from in vitro studies were further validated in 30 randomly selected HNSCC patients from the cohort of 104 patients. In HNSCC patients, expression of TRF2 and p38 phosphorylation was checked by WB analysis in tumors and respective cut-margin tissues (Fig. 4a and Supplementary Figure S3). The percentage of expression of TRF2 and p38 phosphorylation in the tumor tissues were calculated with respect to their cut-margin counterparts. The p38 phosphorylation levels were normalized with total p38 protein expression and expression of TRF2 were normalized with GAPDH expression. Pearson correlation analysis showed a positive correlation between p-p38 and TRF2 protein expression in 60% of the cases (Pearson *r* = 0.63 and *p* = 0.005) (Fig. 4b, c).

Furthermore, association of p-p38 with telomeric TRF2 was confirmed by co-immunoprecipitation assay in HNSCC patient tumor lysates followed by immunoblotting with anti-TRF2, anti-p-p38 and anti p38 antibodies (Fig. 4d–f). Co-IP studies showed interaction of TRF2 with p-p38 and p38.

**Fig. 5:**
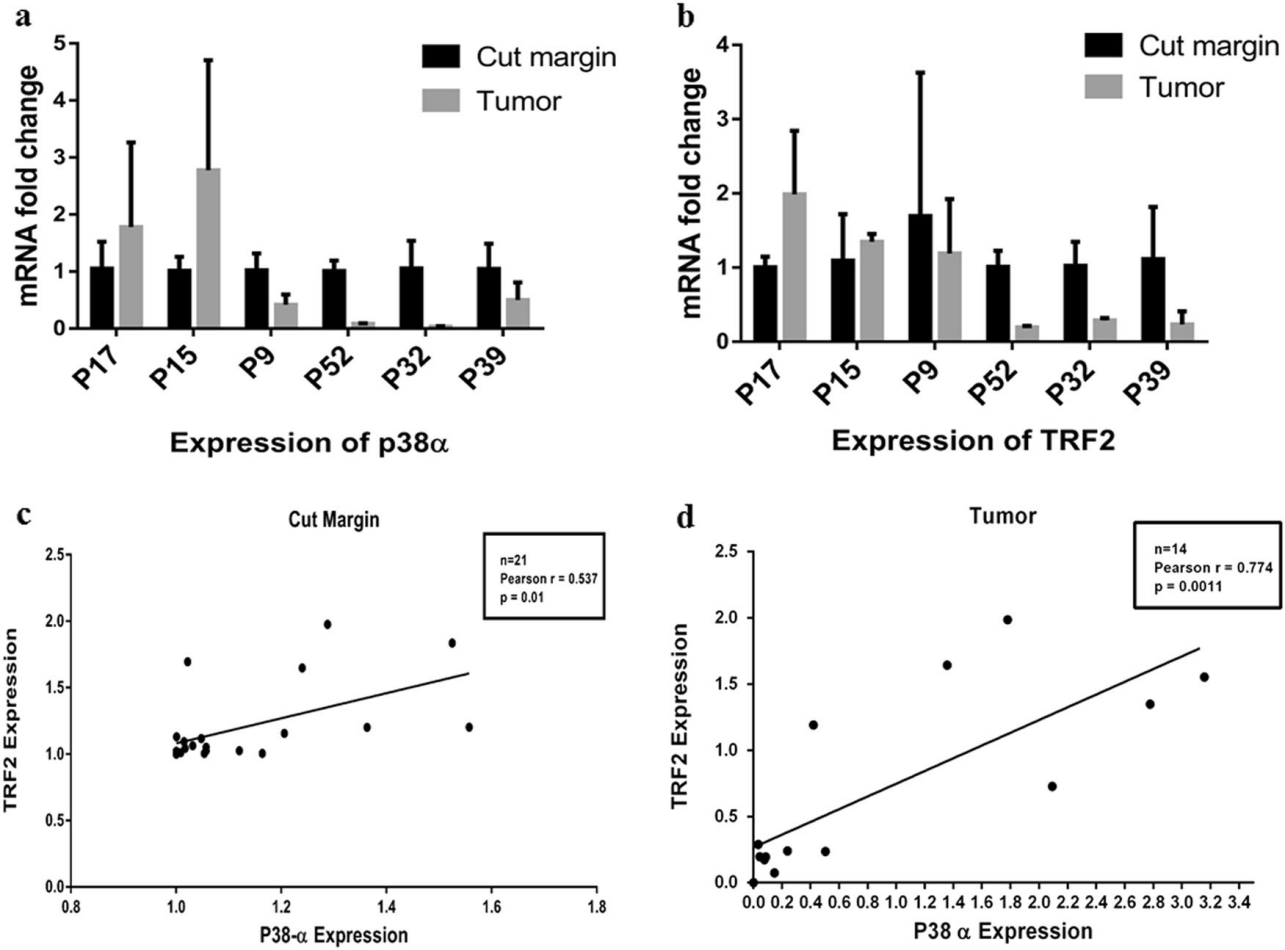
Quantitative real time PCR analysis of p38α and TRF2 expression in HNSCC patient samples. **a**, **b** Quantitative real time PCR analysis of gene expression of p38α and TRF2 for cut margin and tumor tissues of HNSCC cases. *Y*-axis represents fold change in mRNA expression. **c** Twenty-one cases showing a positive correlation for the gene expression of TRF2 and p38α (*p* < 0.05) in cut margin tissues. **d** Fourteen cases showing a positive correlation for the gene expression of TRF2 and p38α (*p* < 0.005) in tumor tissues

Differential gene expression analysis of TRF2 and p38α also revealed a positive correlation between TRF2 and p38α in cut margins of 21 HNSCC cases (Pearson *r* = 0.537 and *p* = 0.01) (Fig. 5a–c and Supplementary Figure S4). In 66.66% cases, the expression of TRF2 gene was found to be positively correlated with the expression of p38α gene (Pearson *r* = 0.774 and *p* = 0.0011) in the tumor tissues (Fig. 5d).

### Presence of disordered regions in TRF2 and p38 proteins

**Fig. 6:**
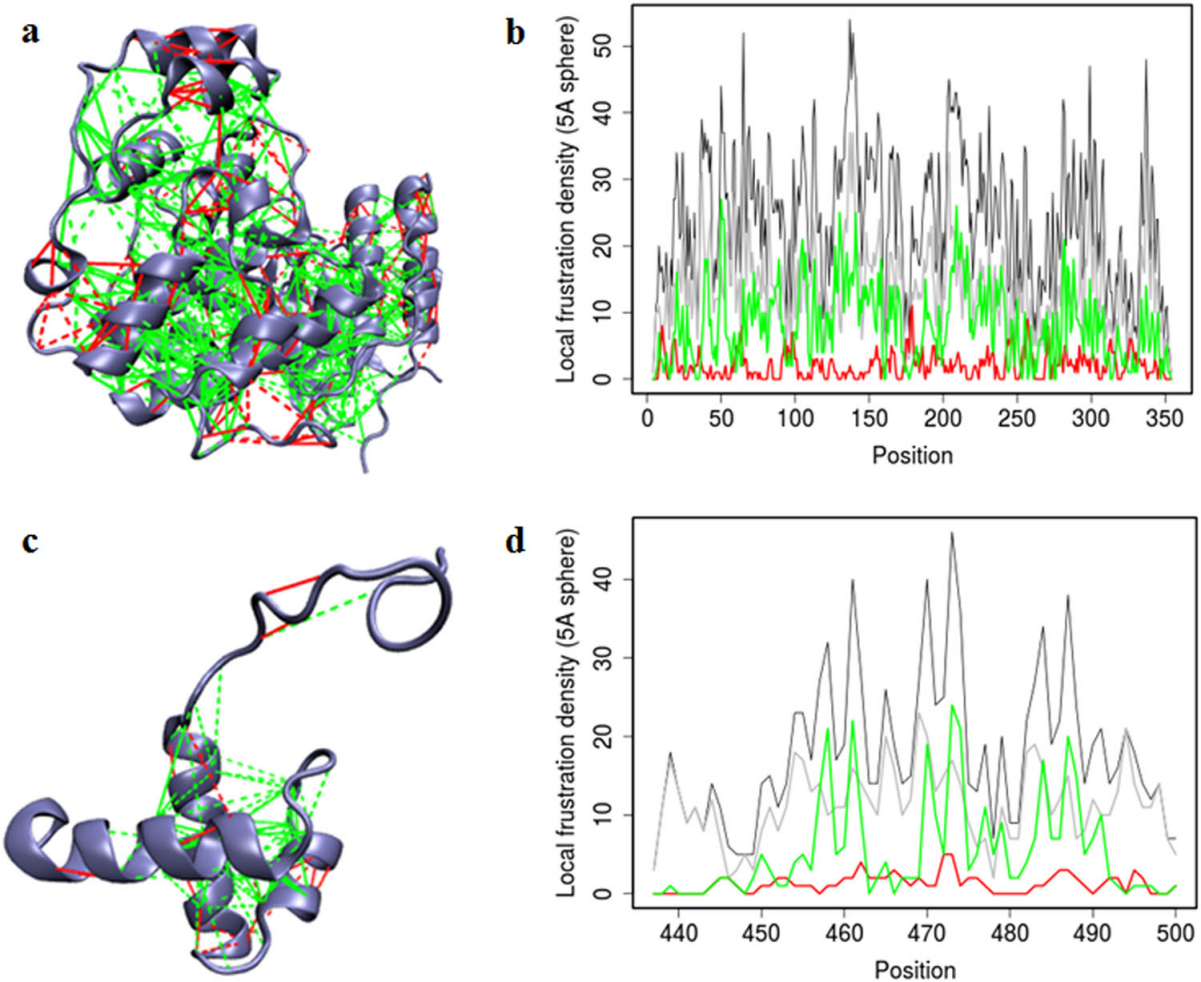
Disordered regions of proteins involved in the potential interactions of TRF2 and p38. Localized frustration and minimally frustrated networks in the protein structures p38 (PDB ID: 1BL6) and TRF2 (PDB ID: TRF2). **a**, **c** The protein backbone is displayed as blue ribbons, the direct inter-residue interactions with solid lines and the water-mediated interactions with dashed lines. Minimally frustrated interactions are shown in green, highly frustrated contacts in red, neutral contacts are not drawn. **b**, **d** Projection of local frustration distribution in the amino acid sequences of proteins. The number of contacts within 5 A° of the C-α of each residue is plotted, as classified according to their frustration index

Our findings were validated by understanding the computational protein–protein interactions between p38-TRF2 interacting protein heterocomplexes. In our study, we applied both Frustratometer method and conserved domain analysis method to estimate protein–protein interactions^24^. We quantified and localized frustrations using frustratometer tool to designate the disordered regions of p38 and TRF2 receptor complex which were involved in interactions at molecular and cellular level (Fig. 6). On analysing p38 and TRF2 proteins, a stretch of highly frustrated regions at amino acid positions: 163–190, 326–352, 295–325 in p38 and 442–447 and 472–477 in TRF2 were observed, shown in Fig. 6b, d respectively. A density map is also shown in Fig. 6b, d, which represents the projections of contacts in sequence space^25^ and regions with high frustrations located at the ends (highlighted with red color, Fig. 6b, d). It has been reported earlier that the highly frustrated regions of the protein correspond to physiologically relevant regions and could play a significant role in protein–protein binding during cellular interactions^26^.

### The strong affinity and stability of p38–TRF2 protein complex

Five conserved domains were found each in p38 and TRF2 proteins respectively. Serine/threonine protein kinase domain of p38 is located between the amino acid residue positions 25–369 and 43–364, whereas DNA binding domains of TRF2 lie in the regions corresponding to residues 489–537, 489–538, and 490–538 positions.

**Fig. 7:**
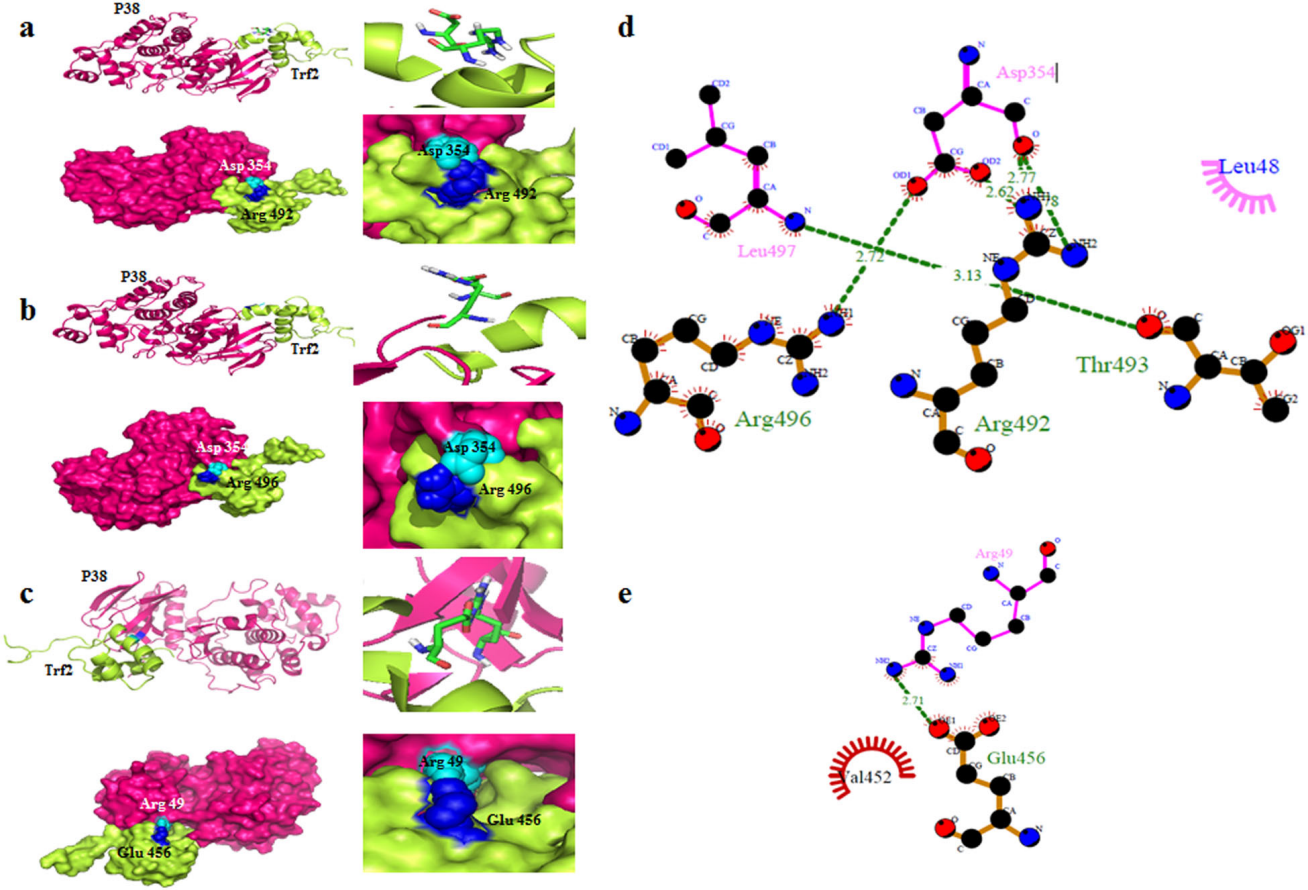
p38-TRF2 protein complex has strong affinity and stability. **a**–**c** The cartoon representation, surface and enlarged view of unphosphorylated p38 (PDB ID:1BL6) and TRF2 (PDB ID: 1VF9) docked complex representing their binding sites involved in protein–protein interactions. **d**–**e** DIMPLOT program generated two-dimensional plots representing hydrogen bonds and other non-bonded interactions between the interacting amino acid residues of p38-TRF2 complex (amino acid residues highlighted in pink and green color respectively)

From docking analysis, we identified polar amino acid residues Asp-354 of p38 and residues Arg-492 and Arg-496 of TRF2 to be involved in the formation of protein-protein complex (Fig. 7a, b). In addition, another amino acid residue Arg-49 of p38 was also found interacting with residue Glu-456 of TRF2 (Fig. 7c). The residues within the same interacting domains resulted in a strong domain–domain interaction between these two proteins. Moreover, the residue positions with strong interactions also showed consensus with Frustratometer density graph, which further supported our findings. The most relevant docked model of p38-TRF2 is shown in cartoon and surface view (Fig. 7a–e). We further analyzed the interactions across the interface of p38-TRF2 complex that were facilitated by hydrogen bonds and hydrophobic interactions^27,28^. We found Asp-354 of p38 to be involved in favorable interactions with Arg-492 and Arg-496 of TRF2. Similar interactions were also observed between Arg-49 of p38 with Glu-456 of TRF2. Hence, these results reveal that p38 could have physical one-to-one interactions with TRF2. The probable amino acid residues of p38 that may be involved in such interactions with TRF2 at the binding interface are shown in Fig. 7d, e.

### Comparison of binding affinity, stability and conformational differences between unphosphorylated (PDV ID: 1BL6) and phosphorylated (PDB ID: 3PY3) structures of p38 MAP kinase with TRF2

**Fig. 8:**
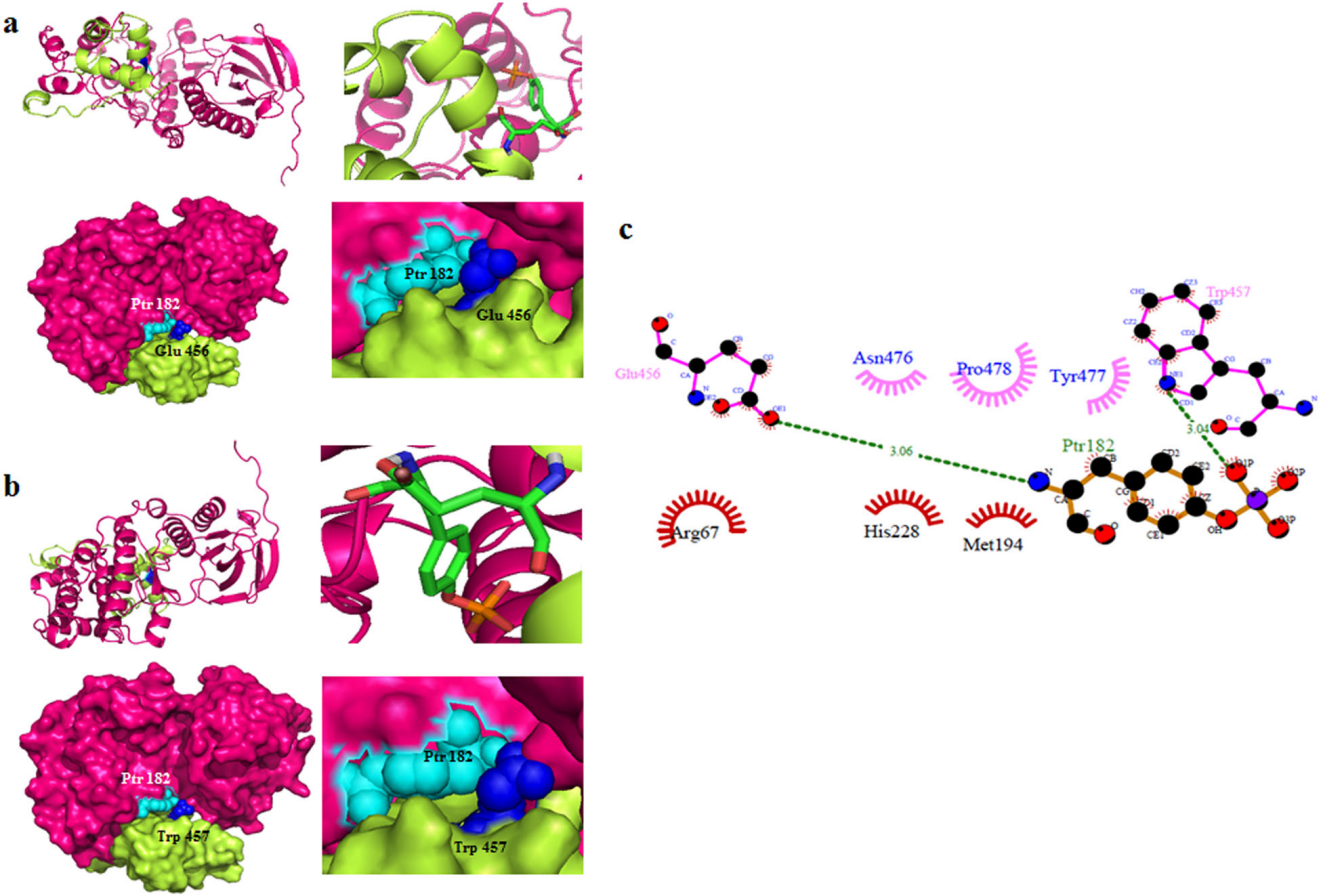
Comparison of the binding affinity, stability and conformational differences between unphosphorylated (PDB ID: 1BL6) and phosphorylate1d (PDB ID: 3PY3) structures of p38 MAP kinase with TRF2 (PDB ID: 1VF9). **a**, **b** The cartoon representation of phosphorylated p38 and TRF2 docked complex showing their binding sites. **c** DIMPLOT program generated two-dimensional plots representing hydrogen bonds and other non-bonded interactions between the interacting amino acid residues of the p38–TRF2 complex

Activation of p38 MAPK involves phosphorylation at amino acid position Thr180 and Tyr182^28^. In this study, both active and inactive forms of p38 MAPK were found to be interacting with TRF2. Thus, we analyzed the conformational change that may occur at binding sites and compared the two most probable p38 conformations interacting with TRF2. In detail, the docked p38 and TRF2 complexes in their inactive (Fig. 7a–c) and active forms (Fig. 8a, b) were analyzed. The phosphorylated amino acid residues, phosphotyrosine Ptr-182 of p38 (PDB ID: 3PY3) displayed interactions with Trp-457 and Glu-456 of TRF2 (Fig. 8a and b). In the unphosphorylated state, protein-protein interactions involving amino acid residues Arg-492 and Arg-496 of TRF2 and Asp354 of p38 (PDB ID: 1BL6) were observed (Fig. 7a–e). The comparative interaction interface analysis showed that phosphorylation may have increased the interaction abilities of amino acid residues present in the binding site of p38 MAPK as compared to unphosphorylated protein. Hydrogen and hydrophobic interactions of the phosphorylated and unphosphorylated p38 estimated the interfaces with which p38 might be interacting with the TRF2. Dimplot analysis also showed interactions of these residues at similar positions as shown in Fig. 8c.

### Molecular dynamics (MD) simulations on protein–protein complexes

The stability of wild type p38 protein and phosphorylated p38 protein in complex with TRF2 protein were monitored by calculating root mean square deviation of C-alpha atoms during MD simulations (Supplementary Figure S5 and S6). p38 protein was found to display a slightly lower value of RMSD (1.7 Å) in the phosphorylated form with respect to the wild type (2.0 Å).

### Interaction network analysis

**Fig. 9:**
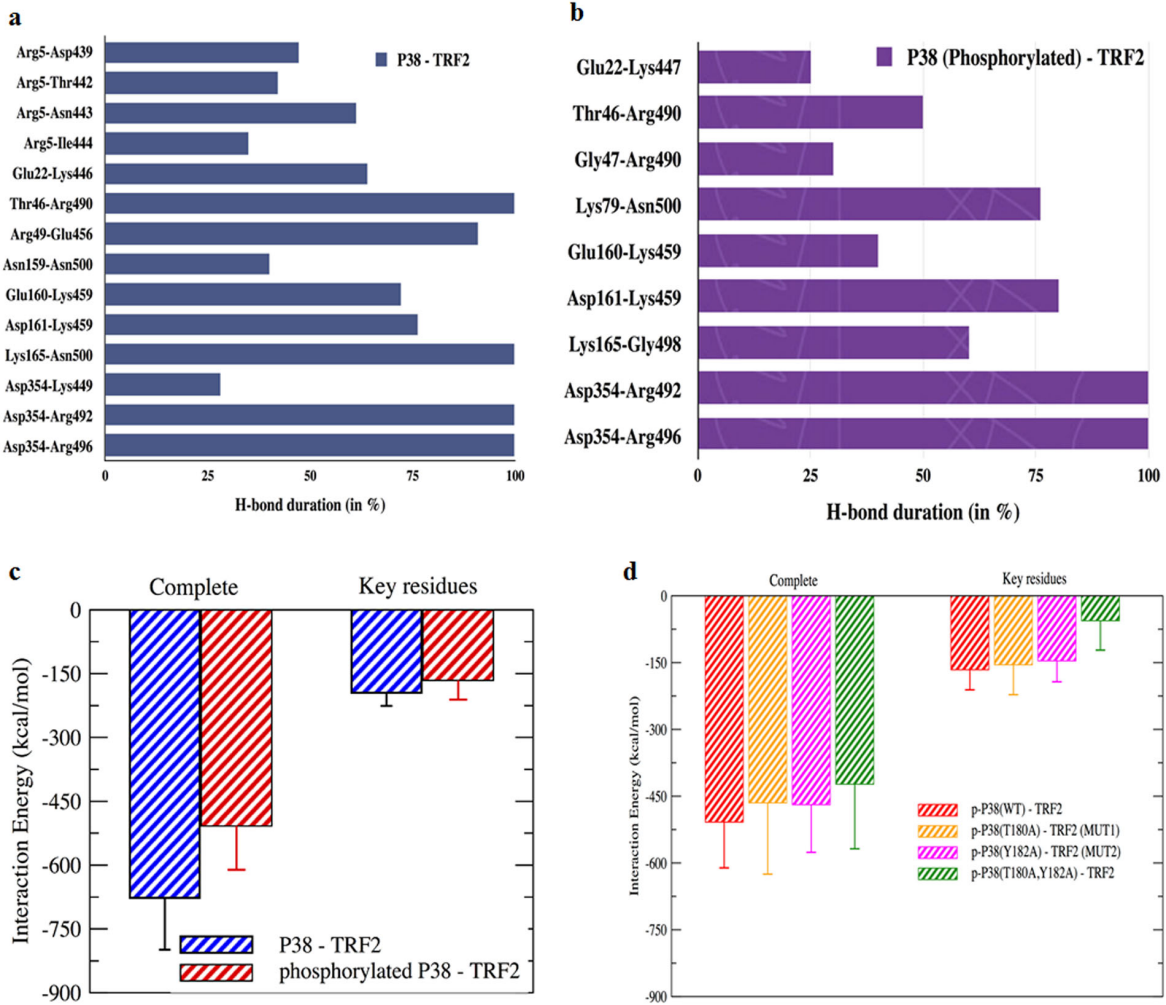
Molecular dynamics (MD) simulations showing hydrogen bond interaction between p38 and TRF2 residues and interaction energy estimation. **a** Wild type simulation. **b** Phosphorylated simulation. **c** The term “complete” corresponds to all the residues of p38 and TRF2, while “key residues” corresponds to interaction energy calculated between selected residue D354 of p38 protein and residues R492 and R496 of TRF2 protein. The interaction energy corresponds to the non-bonded energy values comprising of Van der Waals and electrostatic energy. **d** Energy plot for wild type p-p38 and p-p38 mutants in complex with TRF2

Hydrogen-bond (H-bond) interactions were analysed between residues of TRF2 and residues of p38 protein in the wild type and phosphorylated form (Fig. 9a, b). We found 14 persistent H-bond interactions between residues of wild type p38 protein and TRF2 protein complex, while 9 persistent H-bond interactions between residues of phosphorylated p38 protein and TRF2 protein complex. Conserved p38-TRF2 H-bond interaction pairs were observed between the wild type p38 and p-p38 protein complexes (Asp354–Arg 492, Asp354–Arg 496). Interestingly, only for the wild type complex we found residue Arg-5 of p38 protein to be involved in four persistent H-bond interactions with loop residues of TRF2 protein. Conserved stacking contact between Pro-352 (p38) and (TRF2) Arg-496 in both the wild type and phosphorylated p38 protein simulations were also found. An additional stacking interacting pair His-48 (p38) – (TRF2) Arg-490 was found only in the p-p38 protein complex simulation.

An improved interaction energy value was observed for the wild type p38 protein and TRF2 complex simulation (−677 ± 121 (kcal/mol) with respect to the p-p38 protein–TRF2 complex (−508 ± 108 kcal/mol) simulations (Fig. 9c). However, considering only the key residues (Fig. 9c) a similar value of interaction energy for the wild type- and p-p38-TRF2 protein simulations was found.

To understand the importance of phosphorylation site residue (T180, Y182) on specificity and nature of the interactions between p-p38 and TRF2 protein–protein complexes, in silico mutations of these two residues to alanine was performed. In detail, mutant T180A, mutant Y182A and one double mutant system T180A-Y182A were generated and subsequently subjected to MD simulations.

Interaction energy calculations between p-p38 and TRF2 protein–protein complexes were calculated for mutant simulations and compared with wild type complex simulations.

In general, upon mutation we observed a decrease in interaction energy values with respect to the wild type case, with a greater impact (decrease ~17%) observed for the double mutant system. A similar trend was observed also for the interaction energy calculated considering only the key residues of p-p38 and TRF2 protein–protein complexes. A greater impact of double mutation was found, which resulted in a decrease in the interaction energy value by 65% with respect to the wild type complex simulation (Fig. 9d).

### Silencing of TRF2 and p38 inhibition increases cisplatin sensitivity in SCC-131 cells

**Fig. 10:**
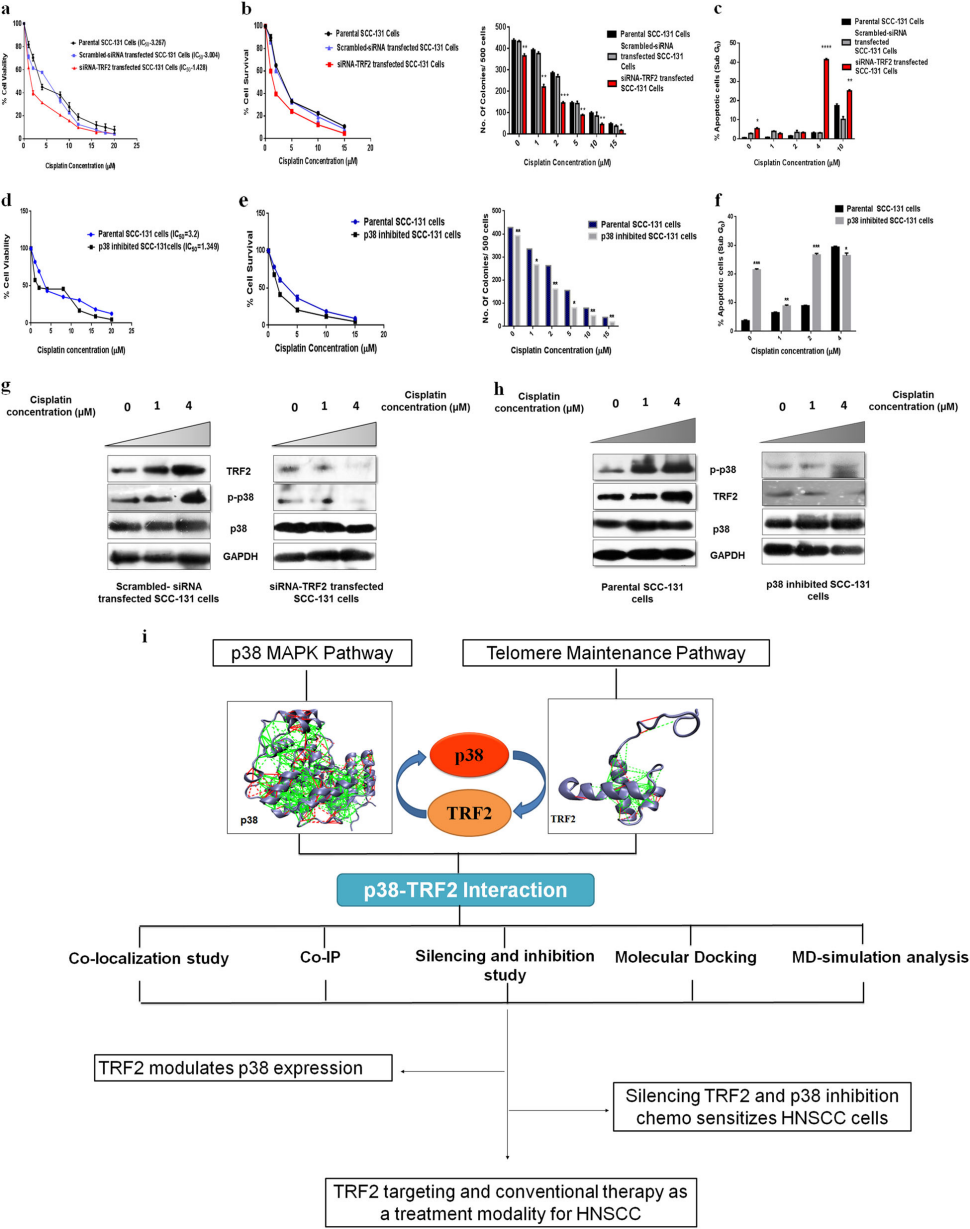
Expression level of TRF2 and p38 phosphorylation modulate chemo-sensitivity of SCC-131 cells towards cisplatin. **a** Cell viability assay of parental cells, scrambled siRNA transfected cells and TRF2 siRNA transfected cells treated with increasing dose of cisplatin showing TRF2 silencing sensitizes cells to cisplatin (IC_50_ = 1.428). **b** Determination of colony forming capacity and cell survival of parental cells, scrambled siRNA transfected cells and TRF2 siRNA transfected cells after cisplatin treatment (1–15 µM) by clonogenic assay. **c** Graphical representation of percentage of apoptotic cells (SubG_0_) in parental cells, scrambled siRNA transfected cells and siRNA TRF2 transfected cells treated with increasing dose of cisplatin. There was a sharp increase in SubG_0_ population at 4 µM concentration after TRF2 silencing. **d** Cell viability assay of parental SCC-131cells and p38 inhibited SCC-131 cells treated with increasing dose of cisplatin showing p38 inhibition sensitizes cells to cisplatin (IC_50_ = 1.349). **e** Determination of cell survival and colony forming capacity of parental SCC-131 cells, and p38 inhibited SCC-131 cells after cisplatin treatment (1–15 µM) by clonogenic assay. **f** Graphical representation of percentage of apoptotic cells (Sub G_0_) in parental SCC-131 cells and p38 inhibited SCC-131 cells treated with increasing dose of cisplatin. There was a sharp increase in SubG_0_ population at 2 µM concentration after p38 inhibition. **g** Representative blots showing reduced expression of TRF2 and p38 phosphorylation in siRNA TRF2 transfected cells as compared to scrambled siRNA transfected cells with increasing cisplatin treatment (0–4 µM). **h** Representative blots showing reduced p38 phosphorylation and TRF2 in p38 inhibited SCC-131 cells as compared to parental SCC-131 cells with increasing cisplatin treatment (0–4 µM). **i** Schematic representation of experimental findings and outcome of the study. Data presented is the mean ± SD of three independent experiments. Statistical significance was determined by two-way ANOVA test (*p *<* 0.05), (**p *<* 0.005), (***p *<* 0.001)

Furthermore, to explore the effects of TRF2 silencing and p38 inhibition in drug sensitivity, cell viablity assay was performed. A two-fold decrease in IC_50_ value was observed for siTRF2 (IC_50_ = 1.428 µM) transfected cells as compared scrambled siRNA transfected cells (IC_50_ = 3.004 µM). Parental SCC-131 cells showed an IC_50_ value of 3.267 µM (Fig. 10a). Silencing of TRF2 also led to dose dependent decrease in colony forming ability and percentage cell survival in SCC-131 cells post cisplatin treatment (1–15 µM) as compared to scramble and parental counterpart (Fig. 10b). Dead live staining also revealed 60.5% cell death in si-TRF2 transfected SCC-131 cells as compared to 36.9% in scrambled siRNA transfected cells and 36.7% in parental cells at a cisplatin concentration of 2 µM (Supplementary Figure S7). A significant increase in early apoptotic population was observed in siTRF2 transfected cells in response to cisplatin at concentration of 4 µM as compared to parental cells and scrambled siRNA transfected cells (Fig. 10c, Supplementary Figure S8 and Table S1).

We next investigated inhibition of p38 on chemosensitivity of HNSCC cells towards cisplatin by cell viability assay. A 2.5-fold decrease in IC_50_ value was observed for p38 inhibited SCC-131 cells (IC_50_ = 1.349 µM) as compared to parental SCC-131 cells (IC_50_ = 3.2 µM) (Fig. 10d). Inhibition of p38 also led to dose dependent decrease in colony forming ability and percentage cell survival of SCC-131 cells post cisplatin treatment (1–15 µM) as compared to parental counterpart (Fig. 10e). Cell cycle analysis revealed a three-fold increase in early apoptotic population in p38 inhibited SCC-131 cells when treated with 2 µM of cisplatin with respect to parental cells (Fig. 10f and Supplementary Figure S9).

We further observed that expression of TRF2 and p38 phosphorylation increased with an increase in cisplatin concentration in scrambled siRNA transfected and parental SCC-131 cells. In contrast the expression of TRF2 and p38 phosphorylation attenuated in siTRF2 transfected SCC-131 and p38 inhibited SCC-131 cells after treatment with increasing concentration of cisplatin (Fig. 10g, h). These results suggested that silencing of TRF2 and inhibition of p38 chemo-sensitizes HNSCC cells towards cisplatin.

## Discussion

Cigarette smoke and tobacco exerts its effect by inducing cytokine production, chronic inflammation and oxidative stress that causes DNA damage and thereby plays an important role in initiation and progression of carcinogenesis^29^. Oxidative stress causes cellular oxidative damage by generating reactive oxygen species (ROS). ROS affects cellular processes such as proliferation, senescence and apoptosis that contribute to the development of cancer^4^. Oxidative stress is one of the major contributors towards dysfunctional telomeres and activation of p38 MAPK pathway. It has been reported that lack of telomere protection due to loss of TRF2 triggers a DNA damage response that involve activation of ataxia telangiectasia mutated (ATM)^30,31^. Activation of ATM is known to activate p38 MAPK pathway^16^. Therefore, in this study we explored the interaction between TRF2 (telomere shelterin component) and p38 MAPK pathway in HNSCC.

We performed in vitro studies of interaction between p-p38 and TRF2 in HNSCC cells. Immunofluorescence assay suggested colocalization of p-p38 and TRF2 in the nucleus of HNSCC cells. Co-immunoprecipitation assay of p-p38 and TRF2 revealed an interaction between these two proteins (Fig. 1).

To better comprehend the molecular interaction between TRF2 and p38 in HNSCC, transient silencing of TRF2 gene was performed. TRF2 silencing lead to decreased co-localization of TRF2 and p-p38 in the nucleus of SCC-131 and CAL 27 cells (Fig. 2f and Supplementary Figure S2f) along with decreased phosphorylation of p38 (Fig. 2a, b and Supplementary figure S2a and b). To validate, we performed Co-IP studies in TRF2 silenced SCC-131 cells. On pull down with TRF2 antibody, significant decrease in phosphorylation of p38 was observed (Fig. 2i). Alternatively, inhibition of p38 decreases co-localization of TRF2 and p-p38 in the nucleus of SCC-131 cells (Fig. 3f) along with decreased TRF2 protein expression and mRNA expression (Fig. 3a, c, k). Thus our present study provides a convincing evidence for the first time that activated form of p38 (p-p38) interacts with TRF2 in HNSCC cells.

We further validated our in vitro findings of p-p38 and TRF2 interaction in HNSCC patient cohort. Positive correlation of expression of these two proteins (Fig. 4a–c) and their gene expression profile (Fig. 5a–d) in HNSCC patient cohort were observed. Co-IP study also revealed the interaction of these two proteins in tumor samples of HNSCC patients (Fig. 4d, e).

In silico analysis and MD simulations on protein–protein complexes were performed to confirm our in vitro findings and patient sample data. In silico analysis revealed a novel interaction between Asp-354 polar residue of p38 with Arg-492 and Arg-496 of TRF2 and amino acid Arg-49 of p38 with Glu-456 TRF2 (Fig. 7a, b). Hence, these results showed that p38 may have strong binding affinity with TRF2. In response to stress p38 MAPK gets activated by phosphorylation at the amino acid position Thr180/Tyr182 and translocate into the nucleus^32^. Molecular interaction studies of these two proteins showed that phosphorylation may increase interaction efficiency of these two proteins in comparison to unphosphorylated p38. This was illustrated by interaction studies that more number of strong interactions and binding interfaces were generated in phosphorylated state exhibited by Ptr182 amino acid residue of p38 with Glu 456 and Trp 457 amino acid residue of TRF2, as compared to unphosphorylated state as shown in Figs. 7, 8. The obtained domains may provide putative target sites to repair DNA damage and ultimately prevent telomere shortening and senescence by overcoming dysfunctional telomeres during stressful condition.

MD simulations on protein–protein complexes further comprehended our findings. In this study, investigation was done to check the dynamics of phosphorylation (p38 protein) to induce substantial changes in proteins interaction network, thereby changing their affinity to the target protein (TRF2). A richer interaction was found in wild type case, thus resulting in a better binding affinity between p38 and TRF2 protein complex (Fig. 9a, b). A reduced affinity of phosphorylated p38 (p-p38) to TRF2 stems from the absence of H-bond interaction between residue Arg-5 of p38 protein and loop residues of TRF2 protein. The simulations studies suggested that though p-p38 has a reduced binding affinity to TRF2 compared to the wild type p38, but both p38 and p-p38 interact with hotspot residues Arg-492 and Arg-496 of TRF2.

Since activation of p38 MAPK involves phosphorylation at amino acid position Thr180/Tyr182^28^. In silico mutations of these two residues to alanine was performed. Energy plot for wild type p-p38 and mutant p-p38 in complex with TRF2 showed a decrease in interaction energy upon mutation of the phosphorylated residues. The decrease was nearly ~17% upon double mutation. Considering the interaction energy values between the key residues the decrease was even more evident with the double mutant showing a decrease by nearly 65% with respect to wild type case.

In human colon cancer, increased TRF2 levels in tumor cells decreases the recruitment and activation of natural killer cells and plays an important role in tumorigenesis^33^. Suppression of TRF2 activates an ATM-dependent DNA damage response pathway that induces apoptosis or senescence^34^. In this respect, we elucidated the impact of TRF2 silencing in chemo-sensitivity of SCC-131 cell line. Cisplatin is the most common chemotherapeutic drug used in treatment of different cancers^35,36^. Treatment with cisplatin results in generation of reactive oxygen species which activate various downstream proteins, in particular, MAPK family proteins^37^. In our study, cell viability assay and clonogenic survival assay revealed that silencing of TRF2 and inhibition of p38 chemo-sensitizes SCC-131 cells when treated with cisplatin. Through cell cycle regulation profile, we determined that there was an increase in early apoptotic population post 4 µM of cisplatin treatment in siTRF2 transfected cells as compared to parental cells and scrambled counterparts (Fig. 10c). Similar result was obtained for p38 inhibited SCC-131 cells. There was a three-fold increase in early apoptotic population in p38 inhibited SCC-131 cells when treated with 2 µM of cisplatin as compared to parental cells (Fig. 10f). A significant decrease in the expression of TRF2 and p38 phosphorylation was observed in siTRF2 transfected and p38 inhibited SCC-131 cells as compared to their controls with increasing cisplatin treatment (Fig. 10g, h). In control cells there was an increase in the expression of TRF2 and p38 phosphorylation with increasing cisplatin concentration. This increase in expression could be due to the stress created by cisplatin. In siTRF2 transfected and p38 inhibited SCC-131 cells the expression of TRF2 and p38 phosphorylation decreased with cisplatin treatment which suggested that TRF2 influences the phosphorylation of p-p38 and vice versa. Hence, silencing of TRF2 and inhibition of p38 might have clinical implication in treatment of HNSCC.

The MAPK family comprises of three major serine/threonine kinase proteins such as p38, Extracellular-signal-regulated kinase (ERK) and Jun amino-terminal kinases (JNK) which are associated with cell growth and differentiation, and are extensively linked to inflammation, apoptosis and cell death^38^. p38 MAPK increases cell survival in cancer cells as a response to DNA damage. Thus, it could be a protective machinery that cells develop to overcome the effect of cytotoxic drugs affecting DNA integrity^16^. Previous reports have demonstrated that inhibition of p38 MAPK sensitizes cancer cells to cisplatin and induce apoptosis by generation of reactive oxygen species^39^.

Our results showed that silencing of TRF2 suppresses the phosphorylation of p38 and inhibition of p38 decreases the expression of TRF2. The schematic representation of outcome of this study was illustrated in Fig. 10i. Thus, targeting TRF2 in combinatorial therapeutics can be a treatment modality for Head and Neck cancer which involves inhibition of p38 MAPK pathway.

## Materials and methods

### Ethic statement

The human sample collection was followed strictly as per institutional ethical board guidelines and conducted according to Helsinki declaration. The study was approved by institutional ethics committee of School of Biotechnology, KIIT University Bhubaneswar. Consent form was duly signed from the patient or their nominees prior to participation in the study.

### Reagents and cell culture

HNSCC cell lines, UPCI-SCC-131, and CAL 27 were maintained in DMEM (HIMEDIA) with 1% antibiotic (100 units of penicillin and 10 mg/ml of streptomycin (HIMEDIA, India), 10% FBS (HIMEDIA, India) and 1% (w/v) of l-glutamine (HIMEDIA, India) in a humidified CO_2_ incubator in 5% CO_2_ in 37 °C. Commercially obtained antibodies and chemicals are mentioned in Supplementary Materials and Methods.

### Patient sample collection

One hundred and four patient tissues with Head and Neck squamous cell carcinoma were acquired at the time of surgical removal of the tumor tissue across the cut margin area and stored accordingly. Voluntary consent forms were signed before each collection of samples.

### Silencing of TRF2 and p38 inhibition in HNSCC cells

TRF2 silencing was performed in SCC-131 and CAL 27 cell lines as per manufacturer’s instructions (Dharmacon) and laboratory established^40^.

For p38 inhibition, cells were seeded at a density of 1 × 10^4^ cells per well. Cells were treated with 10 µM of p38 inhibitor (SB 203580) for 4 h. Additional information is provided in Supplementary Materials and Methods.

### Protein extraction /SDS-PAGE/western blot

To check the expressions of proteins, western blot analysis was carried out for patient tissues and cell line as per laboratory established protocols^1^. Additional information is provided in Supplementary Materials and Methods.

### Immunocytochemistry

Immunocytochemistry assay was performed as per laboratory established protocol^41^. Imaging of signals was done by fluorescence microscope Olympus (BX 61) and images were captured using Image Pro Express software. Additional information is provided in Supplementary Materials and Methods.

### RNA extraction and quantitative real time PCR

RNA extraction and quantitative real time PCR was done as per laboratory established protocols^1^. Additional information is provided in Supplementary Materials and Methods.

### Co-Immunoprecipitation (Co-IP)

Co-immunoprecipitation was performed to analyze the interactions between p-p38 and TRF2 proteins in SCC-131 Head and Neck cancer cells and patient tumor tissue. Additional information is provided in Supplementary Materials and Methods.

### Cell viability assay

MTT Assay was performed with SCC-131 cells post treatment with Cisplatin for 24 h. Additional information is provided in Supplementary Materials and Methods.

### Clonogenic survival assay

Colony formation capacities of SCC-131 cells were determined by using clonogenic survival assay post treatment with Cisplatin for 24 h. Colonies formed were counted using gel documentation system (UVP, Germany). Data were represented as number of colonies formed per 500 cells and percent survival relative to control. Additional information is provided in Supplementary Materials and Methods.

### Dead live staining

Flow cytometric analysis of dead live staining was performed using FACS CANTO II (Becton & Dickinson, CA, USA) on SCC-131 cells post treatment with Cisplatin for 24 h. Analysis of data was done by FACS diva software. Additional information is provided in Supplementary Materials and Methods.

### Cell cycle and apoptosis analysis

Cell cycle based analysis of apoptotic population was performed with SCC-131 cells, scrambled by Flow Cytometry (FACS CANTO II, Becton & Dickinson, CA, USA) post treatment with Cisplatin for 24 h. Analysis of data was done by FACS diva software. Additional information is provided in Supplementary Materials and Methods.

### Protein sequence retrieval

Protein sequences for human p38 (Q16539) (Mitogen-activated protein kinase 14-MAPK14) and TRF2 (Q15554) (Telomeric repeat-binding factor 2-TERF2) were retrieved from ftp server at National Center for Biotechnology Information (NCBI) in the form of FASTA format. Additional information is provided in Supplementary Materials and Methods.

### Homology modeling to obtain 3D structure of p38 and TRF2 protein

To predict three-dimensional structure of p38 and TRF2, PDB structures of p38 (PDB ID: 1BL6) and TRF2 (PDB ID: 1VF9) were submitted to Phyre2 server for homology modeling (http://www.sbg.bio.ic.ac.uk/phyre2)^42^. Additional information is provided in Supplementary Materials and Methods.

### Localization of energetic frustration in protein molecules

To evaluate degree of local frustrations illustrated by spatial local interactions in the two proteins, namely Mitogen-activated protein kinases “p38” (PDB Id: 1BL6) and Telomeric repeat-binding factor 2 “TRF2” (PDB Id: 1VF9), Frustratometer web server (http://www.frustratometer.tk) was used^24^. Additional information is provided in Supplementary Materials and Methods.

### Protein–protein interaction through molecular docking

Molecular docking between p38 and TRF2 were performed using ClusPro 2.0 protein–protein docking server (https://cluspro.bu.edu) with default parameters^43^. Additional information is provided in Supplementary Materials and Methods.

### Molecular dynamics (MD) simulations

The starting structure for p38 (PDB id: 1BL6)–TRF2 (PDB id: 1VF9), protein–protein complex was obtained from docking experiment. The p-p38 protein structure was taken from protein data bank (PDB id: 3PY3). The protein–protein complex between the p-p38 (THR-180, TYR-182) and TRF2 was subsequently obtained from docking. The missing hydrogen atoms in the two protein–protein docked complexes were built using psfgen package of VMD software^44^. Each protein–protein complex system was then immersed in a water box, and subsequently counter ions were added in order to have a neutral system. The initial dimension of the simulation box was 110 × 84 × 76 Å^3^, for a total of ~66.000 atoms. We used TIP3P^45^ parameters for water molecules and Charmm22^46,47^ force-field parameters for protein. Protonation state of the residues was assigned using Propka software^48^. The details about the simulation protocol employed has been described in our previous works^49,50^.

We performed analysis on MD trajectory of 100 ns for the protein-protein complexes under investigation. The stability of protein-protein complexes was evaluated by calculating the root mean square deviation (RMSD) values for the C-alpha atoms of residues during MD simulation. The hydrogen bonded (H-bond) interaction between -protein residues pairs was calculated using a geometrical criterion, with a donor-acceptor cut-off distance of 3.1 Å and donor-hydrogen-acceptor cut-off angle 130 degree^49,51^. H-bonds present for at least 20% of trajectory time length were reported. The aromatic stacking interaction between the -protein–protein residue pairs were calculated using EUCB software^52^ with following geometrical criteria: (i) maximum dihedral angle cut-off parameters between the planar/ring side chains of 30°, (ii) centroid distance cut-off between side chains 5.0 Å, (iii) persistence at least 20% of total simulation time^53,54^. The interaction energy between the two selected groups of residues of p38 protein and TRF2 protein was calculated by evaluating the non-bonded energy values comprising of Van der Waals and electrostatic energy, using the energy plugin of NAMD software^55^.

### Statistical analysis

Statistical analysis was performed using the GraphPad Prism 6 software. Two-way ANOVA was performed for expression variations. Parametric correlations (Pearson’s) were performed between p-p38 and TRF2 protein expression and also between p38α and TRF2 gene expression in HNSCC samples. *p < 0.05, **p < 0.005, and *** *p* < 0.001 was considered to be statistically significant.

## Electronic supplementary material

Revised Supplementary material

Movie 1

Movie 2

## References

[1] Padhi S (2015). Clinico-pathological correlation of β-catenin and telomere dysfunction in head and neck squamous cell carcinoma patients. J. Cancer.

[2] Leelahavanichkul K (2014). A role for p38 MAPK in head and neck cancer cell growth and tumor-induced angiogenesis and lymphangiogenesis. Mol. Oncol..

[3] Lee C, Lee C, Atakilit A, Siu A, Ramos DM (2014). Differential spheroid formation by oral cancer cells. Anticancer Res..

[4] Katakwar P, Metgud R, Naik S, Mittal R (2016). Oxidative stress marker in oral cancer: a review. J. Cancer Res. Ther..

[5] Bianchi-Smiraglia A, Nikiforov MA (2012). Controversial aspects of oncogene-induced senescence. Cell Cycle.

[6] Misri S, Pandita S, Kumar R, Pandita TK (2008). Telomeres, histone code, and DNA damage response. Cytogenet. Genome Res..

[7] Smogorzewska A, de Lange T (2004). Regulation of telomerase by telomeric proteins. Annu. Rev. Biochem..

[8] Griffith JD (1999). Mammalian telomeres end in a large duplex loop. Cell.

[9] Sfeir A, de Lange T (2012). Removal of shelterin reveals the telomere end-protection problem. Science.

[10] van Steensel B, Smogorzewska A, de Lange T (1998). TRF2 protects human telomeres from end-to-end fusions. Cell.

[11] Nakanishi K (2003). Expression of mRNAs for telomeric repeat binding factor (TRF)-1 and TRF2 in atypical adenomatous hyperplasia and adenocarcinoma of the lung. Clin. Cancer Res..

[12] Miyachi K, Fujita M, Tanaka N, Sasaki K, Sunagawa M (2002). Correlation between telomerase activity and telomeric-repeat binding factors in gastric cancer. J. Exp. Clin. Cancer Res..

[13] Pal D, Sharma U, Singh SK, Kakkar N, Prasad R (2015). Over-expression of telomere binding factors (TRF1 & TRF2) in renal cell carcinoma and their inhibition by using SiRNA induce apoptosis, reduce cell proliferation and migration invitro. PLoS ONE.

[14] Bozec A (2009). Combination of sunitinib, cetuximab and irradiation in an orthotopic head and neck cancer model. Ann. Oncol..

[15] Dong C, Davis RJ, Flavell RA (2002). MAP kinases in the immune response. Annu. Rev. Immunol..

[16] Thornton TM, Rincon M (2009). Non-classical p38 map kinase functions: cell cycle checkpoints and survival. Int. J. Biol. Sci..

[17] Wood CD, Thornton TM, Sabio G, Davis RA, Rincon M (2009). Nuclear localization of p38 MAPK in response to DNA damage. Int. J. Biol. Sci..

[18] Schieven GL (2009). The p38alpha kinase plays a central role in inflammation. Curr. Top. Med. Chem..

[19] Liu T (2013). Telomerase reverse transcriptase inhibition stimulates cyclooxygenase 2 expression in cancer cells and synergizes with celecoxib to exert anti-cancer effects. Br. J. Cancer.

[20] Spallarossa P (2009). Doxorubicin induces senescence or apoptosis in rat neonatal cardiomyocytes by regulating the expression levels of the telomere binding factors 1 and 2. Am. J. Physiol. Circ. Physiol..

[21] Ludlow AT, Gratidão L, Ludlow LW, Spangenburg EE, Roth SM (2017). Acute exercise activates p38 MAPK and increases the expression of telomere-protective genes in cardiac muscle. Exp. Physiol..

[22] Huda N, Tanaka H, Mendonca MS, Gilley D (2009). DNA damage-induced phosphorylation of TRF2 is required for the fast pathway of DNA double-strand break repair. Mol. Cell. Biol..

[23] Oosting SF (2016). A comparison of weekly versus 3-weekly cisplatin during adjuvant radiotherapy for high-risk head and neck cancer. Oral Oncol..

[24] Jenik M (2012). *P*rotein frustratometer: a tool to localize energetic frustration in protein molecules. Nucleic Acids Res..

[25] Mendoza-Parra MA, Blum M, Malysheva V, Cholley PE, Gronemeyer H (2016). LOGIQA: a database dedicated to long-range genome interactions quality assessment. BMC Genom..

[26] Ferreiro DU, Hegler JA, Komives EA, Wolynes PG (2007). Localizing frustration in native proteins and protein assemblies. Proc. Natl Acad. Sci. USA.

[27] Patil R (2010). Optimized hydrophobic interactions and hydrogen bonding at the target-ligand interface leads the pathways of drug-designing. PLoS ONE.

[28] Yurtsever Z, Scheaffer SM, Romero AG, Holtzman MJ, Brett TJ (2015). The crystal structure of phosphorylated MAPK13 reveals common structural features and differences in p38 MAPK family activation. Acta Crystallogr. D.

[29] Nair U, Bartsch H, Nair J (2004). Alert for an epidemic of oral cancer due to use of the betel quid substitutes gutkha and pan masala: a review of agents and causative mechanisms. Mutagenesis.

[30] d’Adda di Fagagna F (2003). A DNA damage checkpoint response in telomere-initiated senescence. Nature.

[31] Takai H, Smogorzewska A, de Lange T (2003). DNA damage foci at dysfunctional telomeres. Curr. Biol..

[32] Ben-Levy R, Hooper S, Wilson R, Paterson HF, Marshall CJ (1998). Nuclear export of the stress-activated protein kinase p38 mediated by its substrate MAPKAP kinase-2. Curr. Biol..

[33] Biroccio A (2013). TRF2 inhibits a cell-extrinsic pathway through which natural killer cells eliminate cancer cells. Nat. Cell Biol..

[34] Karlseder J, Broccoli D, Dai Y, Hardy S, de Lange T (1999). p53- and ATM-dependent apoptosis induced by telomeres lacking TRF2. Science.

[35] Prestayko AW, D’Aoust JC, Issell BF, Crooke ST (1979). Cisplatin (cis-diamminedichloroplatinum II). Cancer Treat. Rev..

[36] Kelland LR (2000). Preclinical perspectives on platinum resistance. Drugs.

[37] Malik S (2015). Telmisartan ameliorates cisplatin-induced nephrotoxicity by inhibiting MAPK mediated inflammation and apoptosis. Eur. J. Pharmacol..

[38] Wada T, Penninger JM (2004). Mitogen-activated protein kinases in apoptosis regulation. Oncogene.

[39] Pereira L, Igea A, Canovas B, Dolado I, Nebreda AR (2013). Inhibition of p38 MAPK sensitizes tumour cells to cisplatin-induced apoptosis mediated by reactive oxygen species and JNK. EMBO Mol. Med..

[40] Saha A (2017). Role of TRF2 in efficient DNA repair, spheroid formation and cancer stem cell maintenance. Oncomedicine.

[41] Saha A, Shree Padhi S, Roy S, Banerjee B (2014). HCT116 colonospheres shows elevated expression of hTERT and β-catenin protein—a short report. J. Stem Cells.

[42] Kelley LA, Mezulis S, Yates CM, Wass MN, Sternberg MJE (2015). The Phyre2 web portal for protein modeling, prediction and analysis. Nat. Protoc..

[43] Comeau SR, Gatchell DW, Vajda S, Camacho CJ (2004). ClusPro: a fully automated algorithm for protein-protein docking. Nucleic Acids Res..

[44] Humphrey W, Dalke A, Schulten K (1996). VMD: visual molecular dynamics. J. Mol. Graph..

[45] Jorgensen WL, Chandrasekhar J, Madura JD, Impey RW, Klein ML (1983). Comparison of simple potential functions for simulating liquid water. J. Chem. Phys..

[46] MacKerell AD (1998). All-atom empirical potential for molecular modeling and dynamics studies of proteins †. J. Phys. Chem. B.

[47] Mackerell AD, Feig M, Brooks CL (2004). Extending the treatment of backbone energetics in protein force fields: limitations of gas-phase quantum mechanics in reproducing protein conformational distributions in molecular dynamics simulations. J. Comput. Chem..

[48] Rostkowski M, Olsson MH, Søndergaard CR, Jensen JH (2011). Graphical analysis of pH-dependent properties of proteins predicted using PROPKA. BMC Struct. Biol..

[49] Kumar A (2015). Dynamical insights into the differential characteristics of Mycobacterium avium subsp. paratuberculosis peptide binding to HLA-DRB1 proteins associated with multiple sclerosis. New J. Chem..

[50] Kumar A, Delogu F (2017). Dynamical footprint of cross-reactivity in a human autoimmune T-cell receptor. Sci. Rep..

[51] Kumar A (2014). Antigenic peptide molecular recognition by the DRB1-DQB1 haplotype modulates multiple sclerosis susceptibility. Mol. Biosyst..

[52] Tsoulos IG, Stavrakoudis A (2011). Eucb: a C++ program for molecular dynamics trajectory analysis. Comput. Phys. Commun..

[53] Kumar A, Cocco E, Atzori L, Marrosu MG, Pieroni E (2013). Structural and dynamical insights on HLA-DR2 complexes that confer susceptibility to multiple sclerosis in Sardinia: a molecular dynamics simulation study. PLoS ONE.

[54] Balaraju T (2013). Aromatic interaction profile to understand the molecular basis of raltegravir resistance. Struct. Chem..

[55] Phillips JC (2005). Scalable molecular dynamics with NAMD. J. Comput. Chem..

