# Supplementary material for: p38 MAPK pathway and its interaction with TRF2 in cisplatin induced chemotherapeutic response in head and neck cancer
Source: Oncogenesis. 2018 Jul 9;7(7):53. doi: 10.1038/s41389-018-0062-6 (PMC6036057; doi:10.1038/s41389-018-0062-6)
Supplement: Supplementary file 1 — Revised Supplementary material [file 41389_2018_62_MOESM1_ESM.docx]

**p38 MAPK pathway and its interaction with TRF2 in cisplatin induced chemotherapeutic response in head and neck cancer**

**Shomereeta Roy^1#^, Souvick Roy^1#^, Madhabananda Kar^2^, Shweta Thakur^3^, Yusuf Akhter^4^, Amit Kumar^5,6^, Francesco Delogu^5^, Swatishree Padhi^1^, Arka Saha^1^, Birendranath Banerjee^1*^**

^1^Molecular Stress and Stem Cell Biology Group, School of Biotechnology, KIIT University, Bhubaneswar, Odisha-751024, India.

^2^Professor and Head, Department of Surgical Oncology, All India Institute of Medical Sciences (AIIMS), Bhubaneswar, Odisha-751019, India.

^3^Centre for Computational Biology and Bioinformatics, School of Life Sciences, Central University of Himachal Pradesh, Shahpur, Himachal Pradesh-176206, India.

^4^Department of Biotechnology, Babasaheb Bhimrao Ambedkar University, Vidya Vihar, Raebareli Road, Lucknow, Uttar Pradesh 226025, India.

^5^ Departments of Mechanical, Chemical and Materials Engineering, University of Cagliari, via Marengo 2, 09123 Cagliari, Italy.

^6^ Biosciences Sector, Center for advanced study research and development in Sardinia (CRS4), Loc. Piscina Manna, 09010 Pula, Italy.

**Correspondence^*^:** Birendranath Banerjee, Group leader, Molecular stress and stem cell biology group, KIIT School of Biotechnology, KIIT University, Bhubaneshwar-751024, Odisha, India: E-mail: bnbanerjee@kiitbiotech.ac.in, phone: +91- 9090840042. Fax: 0674-2378776.

**^#^** Authors contributed equally.

**Supplementary Figure and Table legends**

**Supplementary Figure S1.** TRF2 and p-p38 co-localizes in CAL 27 cells (a) Representative immunofluorescence images of CAL 27 cells co-stained for TRF2 and p-p38. Merged images indicate co-localization. DAPI was used to visualize nuclear DNA. (b) Corrected Total Cell Fluorescence showing the expression of TRF2 and p-p38 in CAL 27 cells

**Supplementary Figure S2.** TRF2 silencing decreases p38 phosphorylation in CAL 27 cells**.** Cells were transfected with scrambled siRNA and siRNA TRF2 for 48 hours. (a) Representative blots showing decreased expression of TRF2 and p38 phosphorylation in whole cell extracts after silencing TRF2. (b, c) Relative p-p38 and TRF2 protein expression in whole cell extracts post TRF2 silencing. (d) Representative immunofluorescence images of cells showing decreased expression of TRF2 and p38 phosphorylation after TRF2 silencing. (e) Corrected Total Cell Fluorescence showing significantly decreased expression of TRF2 and p38 phosphorylation in TRF2 silenced CAL 27 cells. (f) Representative immunofluorescence images of cells showing decreased co-localisation of TRF2 and p-p38 in TRF2 silenced cells. (g) Corrected Total Cell Fluorescence showing decreased co-localisation of TRF2 and p-p38 in TRF2 silenced cells. (h) Quantitative Real Time PCR analysis showing decreased expression of TRF2 gene after TRF2 silencing. (i) Quantitative Real Time PCR analysis showing no effect in the expression p38α gene after TRF2 silencing. Y-axis represents fold change in mRNA expression. Data presented is the mean $\pm$SD of three independent experiments. Statistical significance was determined by two way ANOVA test (**p* < 0.05), (***p* < 0.005).

**Supplementary Figure S3.**Western blot analysis to show the expression of TRF2 and p38 phosphorylation of 30 HNSCC patient samples in tumor and their respective cut margin tissues.

**Supplementary Figure S4.** Quantitative Real Time PCR analysis to show the expression of p38α and TRF2 in 21 HNSCC patient samples.

**Supplementary Figure S5**. Root mean square deviation plot of Cα atoms with respect to the initial structure during the simulation. In (a) for p38 protein shown in red, in (b) for TRF2 in green and without loop in pink.

**Supplementary Figure S6**. RMSD plot of Cα atoms with respect to the initial structure during the simulation. In (a) for p38 protein shown in red, in (b) for TRF2 in green and without loop in pink.

**Supplementary Figure S7.** Dead live staining in parental cells, scrambled siRNA transfected cells and TRF2 siRNA transfected cells, showing increased percentage of dead cells when treated with increasing dose of cisplatin post TRF2 silencing.

**Supplementary Figure S8**. Cell cycle regulation profile of parental cells, scrambled siRNA transfected cells and siRNA TRF2 transfected SCC-131 cells treated with increasing dose of cisplatin.

**Supplementary Figure S9.** Cell cycle regulation profile of parental SCC-131 cells and p38 inhibited SCC-131 cells after treatment with increasing dose of cisplatin (1-4µM).

**Supplementary Table 1**.Tabular representation of percentage of G_0_, G1, S and G2/M cells in parental cells, scrambled siRNA transfected cells and siRNA TRF2 transfected SCC-131 cells treated with increasing dose of cisplatin Data presented here is mean ± SD of three independent experiments

**Supplementary Figure S1**

**
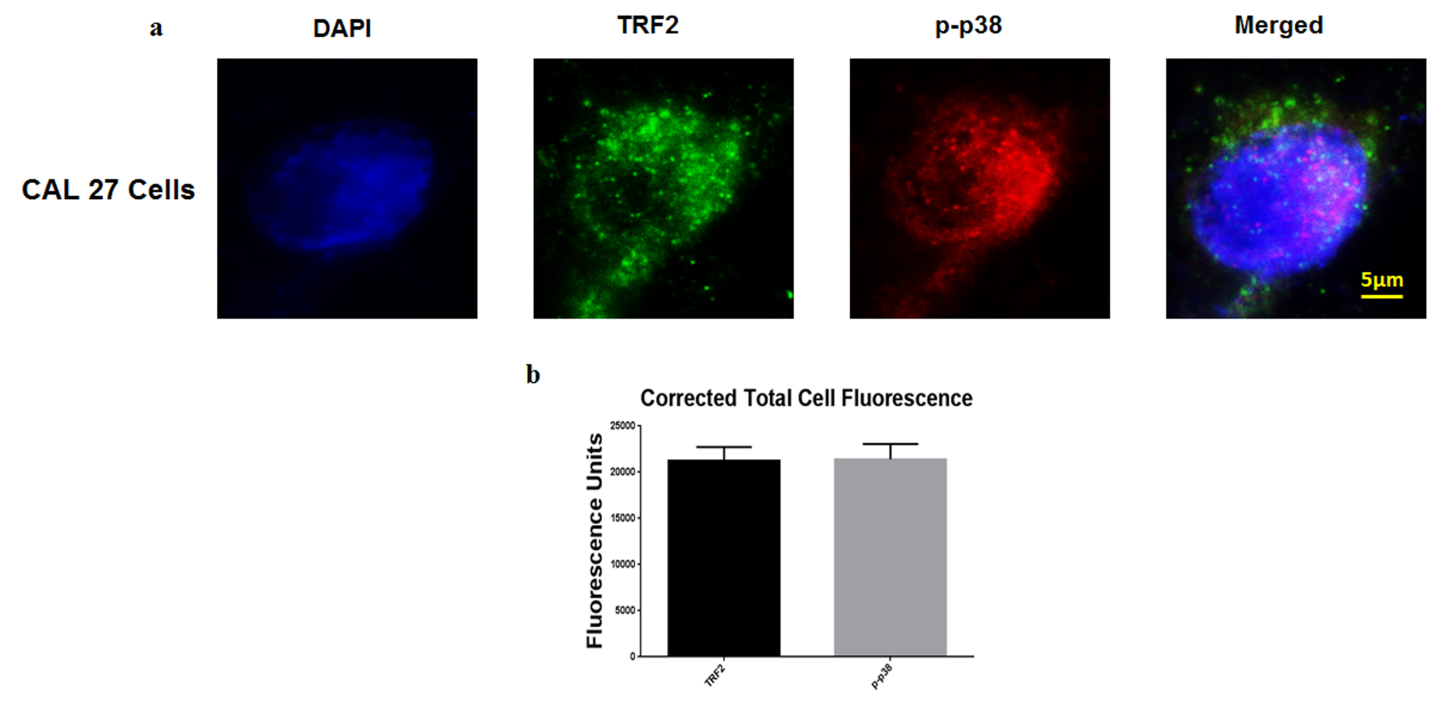
**

**Supplementary Figure S2**

**
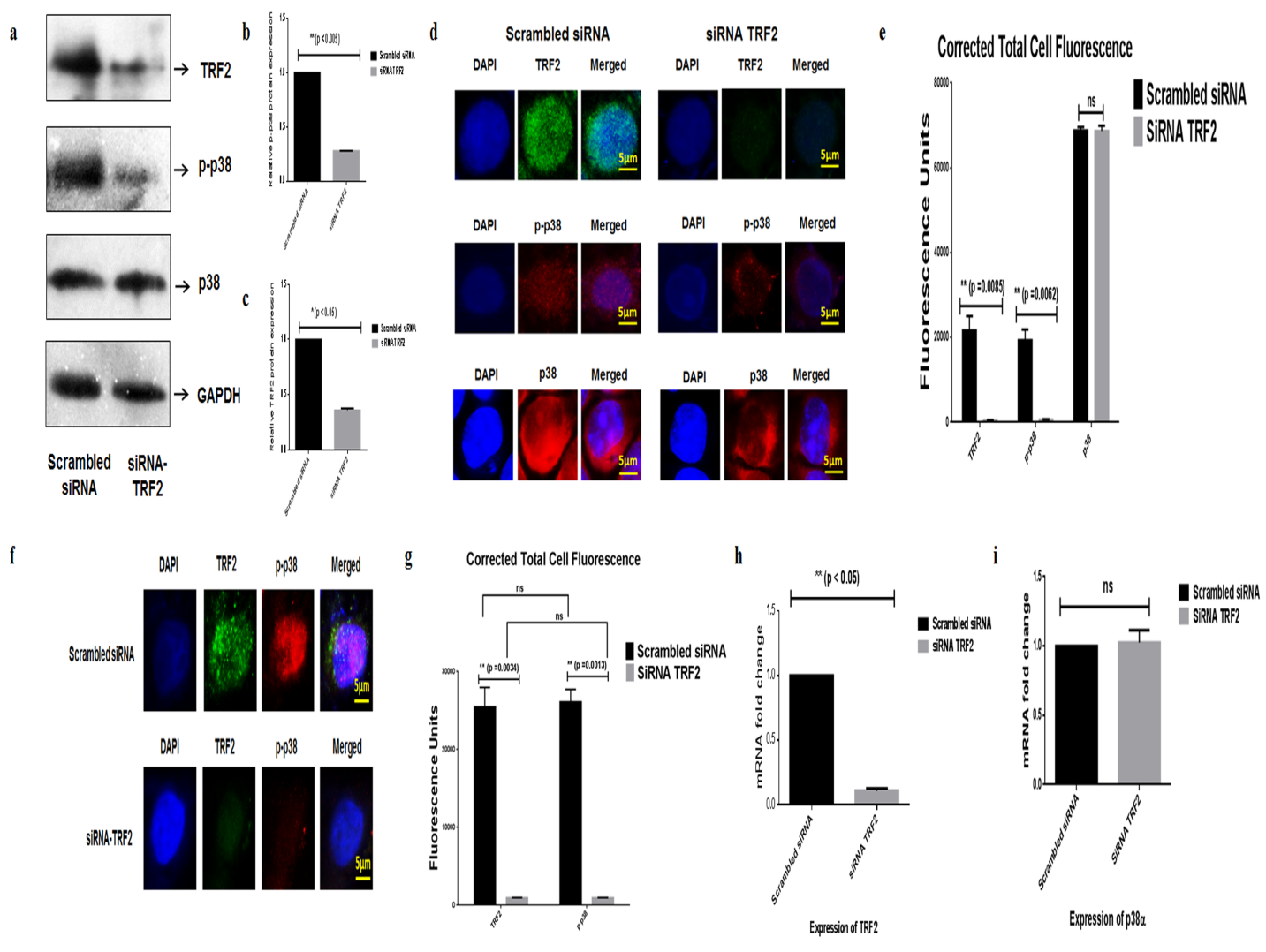
**

**Supplementary Figure S3**

**
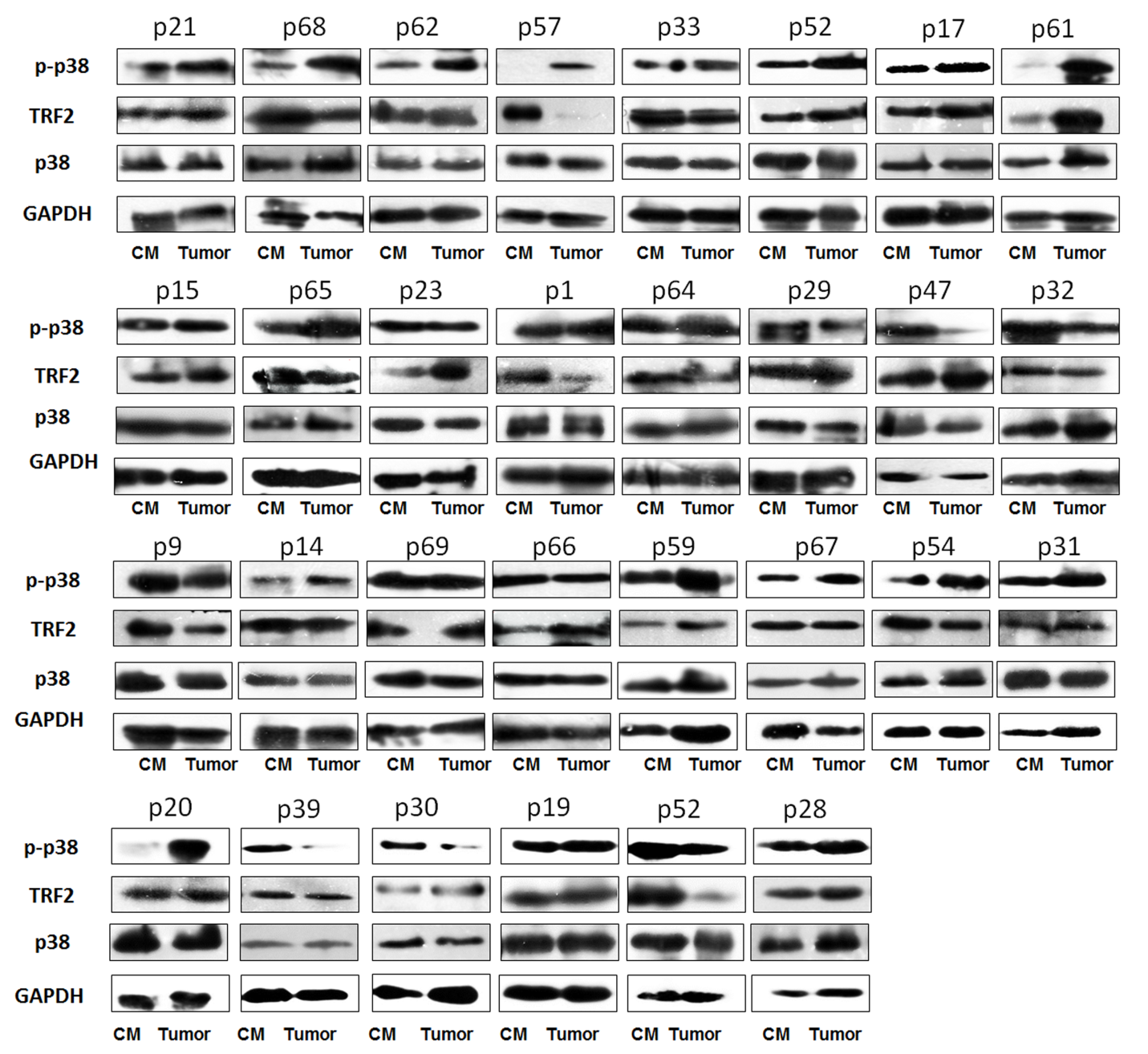
**

**SupplementaryFigure S4**

**
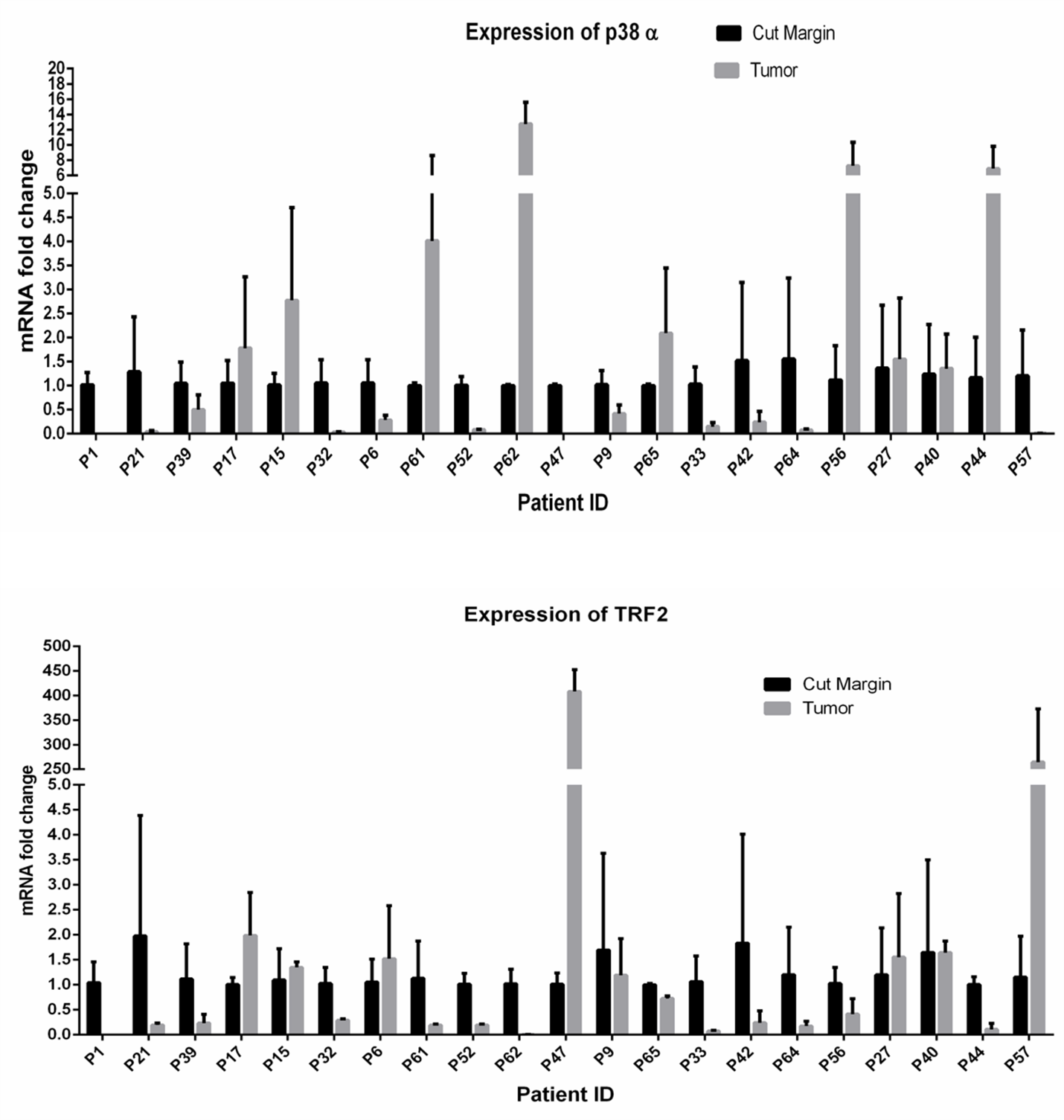
**

**Supplementary Figure S5**


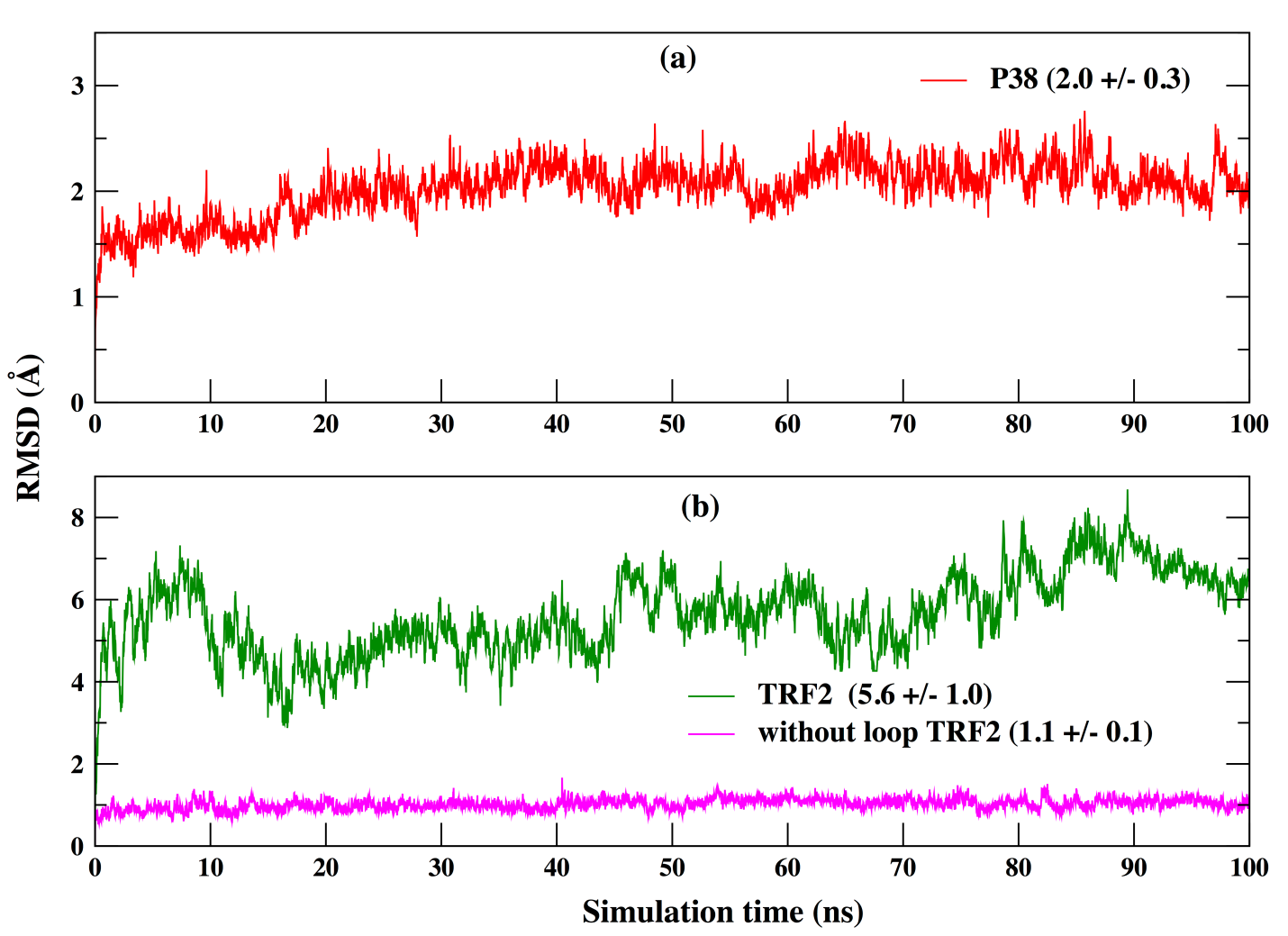


**Supplementary Figure S6**


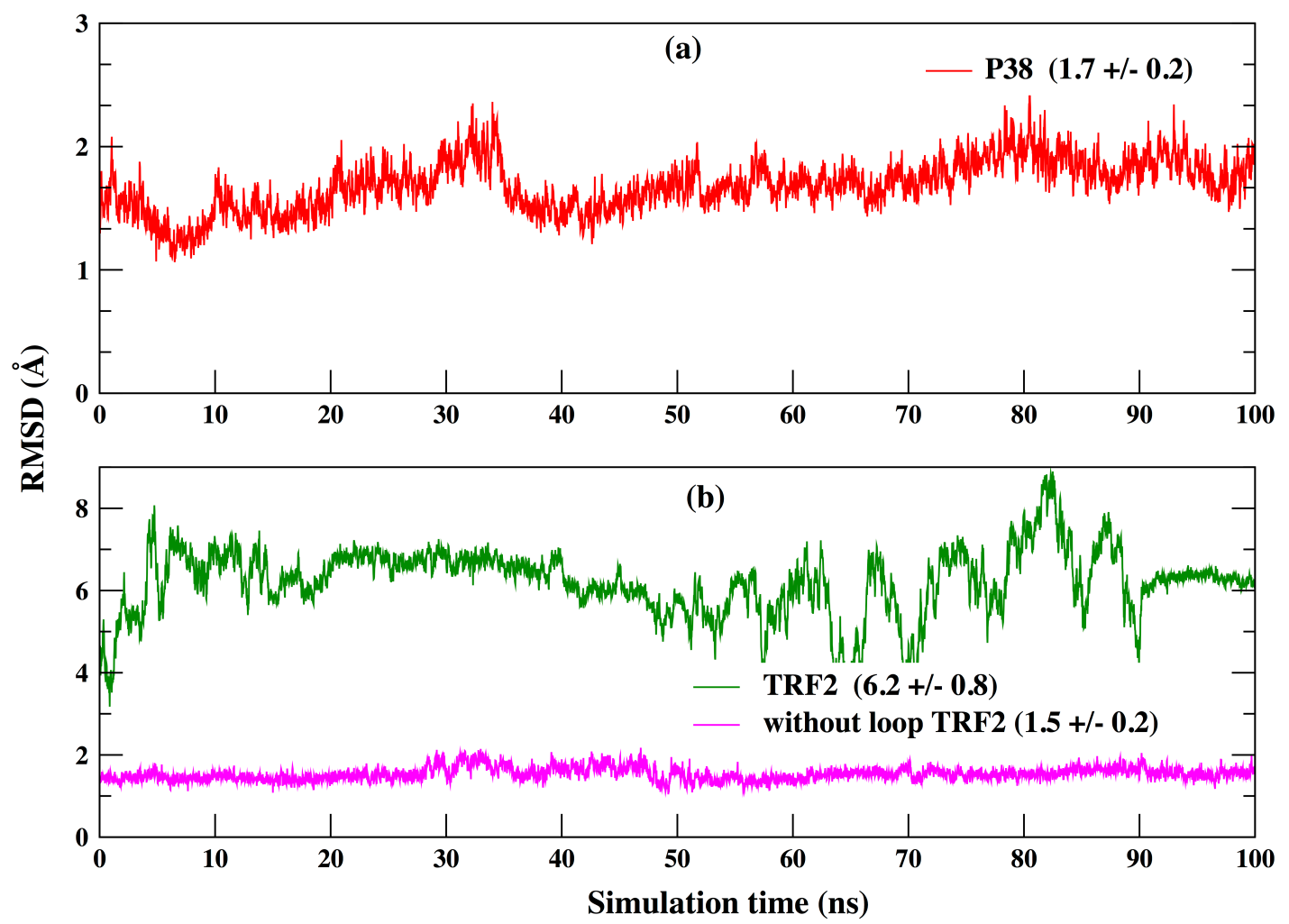


**Supplementary Figure S7**

**
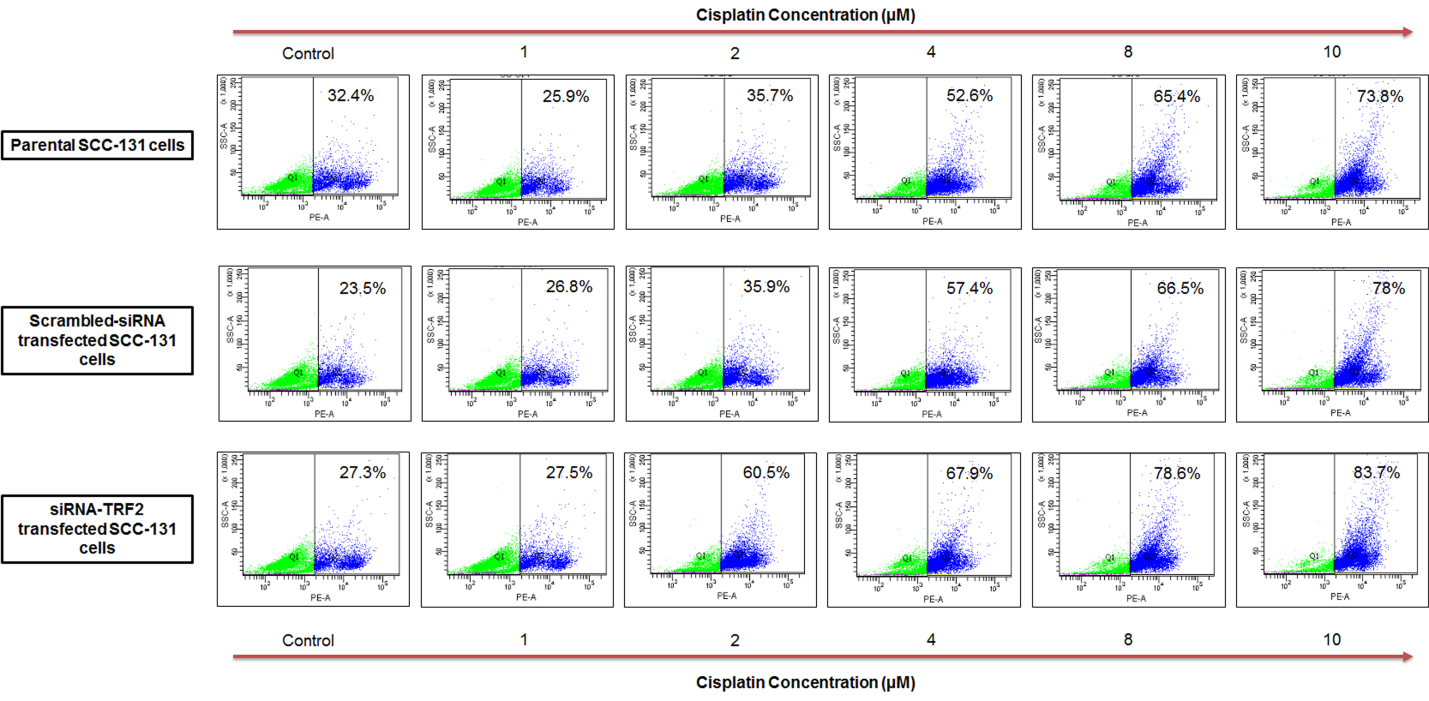
**

**
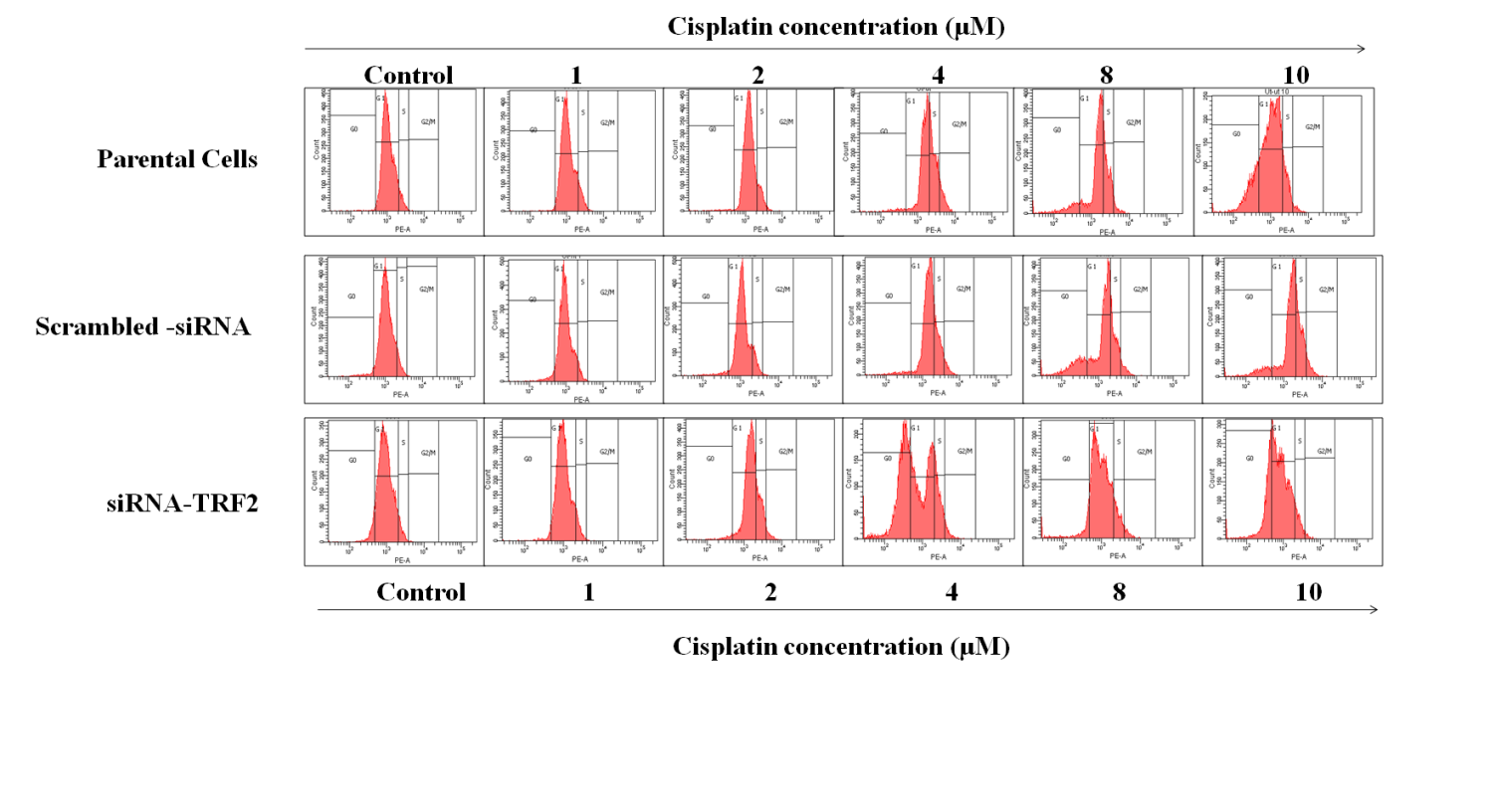
Supplementary Figure S8**

**Supplementary Figure S9**

**
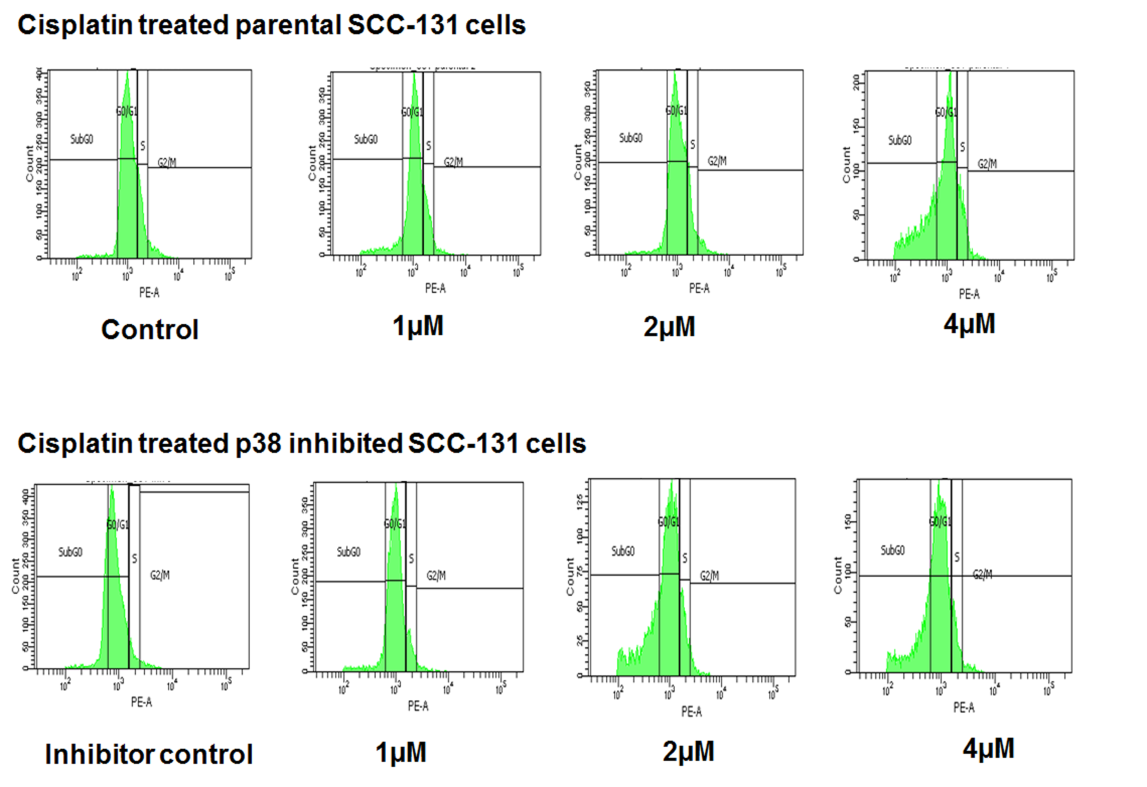
**

**Supplementary Table 1**


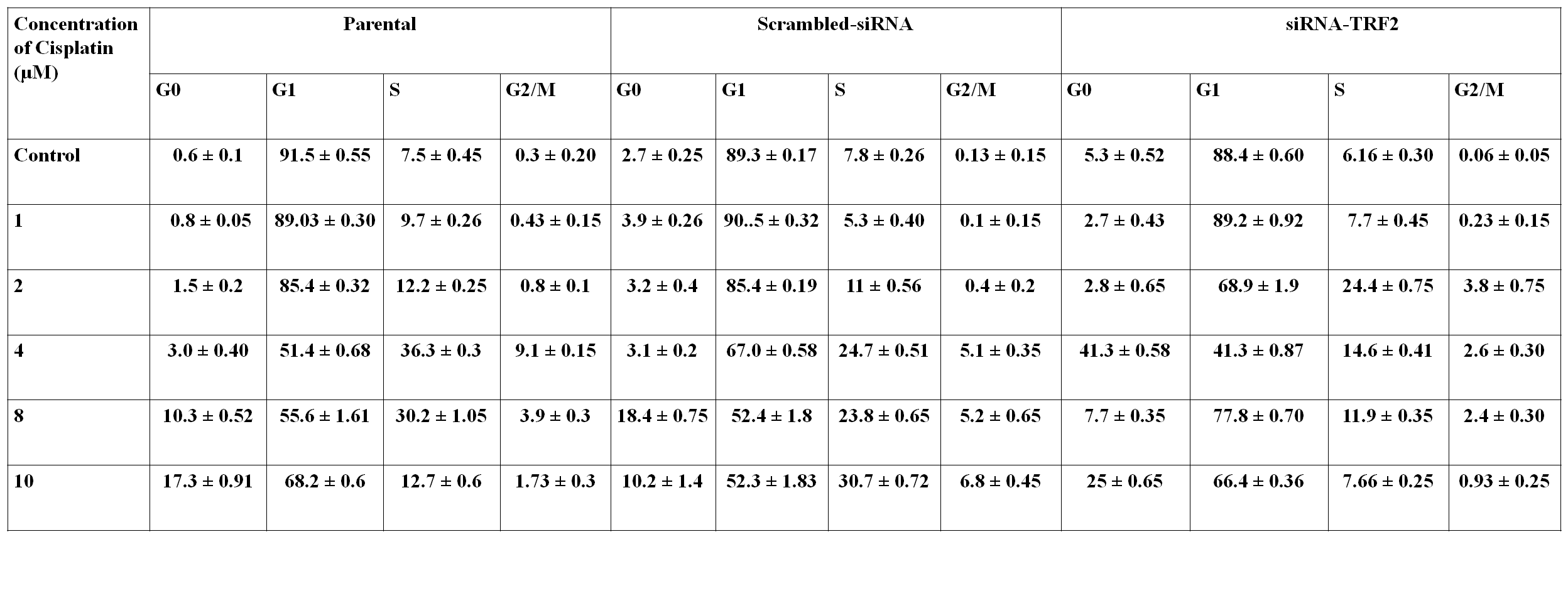


**Materials and methods**

**Reagents and Cell culture**

Human and Neck Squamous Cell Carcinoma (HNSCC) cell lines UPCI-SCC-131 and CAL 27 was maintained in DMEM (HIMEDIA) with 1% antibiotic (100 units of penicillin and 10 mg/ml of streptomycin (HIMEDIA, India),10% FBS (HIMEDIA, India) and 1% (w/v) of L-glutamine (HIMEDIA, India) in a humidified CO_2_ incubator in 5% CO_2_ in 37 °C. p-p38 (Thr180/Tyr182), p-38, TRF2 and IgG isotype, GAPDH antibodies used in the experiments were procured from Cell Signaling Technology (MA, USA), Abcam (Cambridge, United Kingdom) and IMGENEX (India) respectively. p38 inhibitor SB 203580 was procured from Abcam (Cambridge, United Kingdom).

**Patient Sample collection**

Patient tissues with Head and Neck squamous cell carcinoma were acquired at the time of surgical removal of the tumor tissue across the cut margin area and stored accordingly. The study was approved by the institutional ethics committee of the School of Biotechnology and Kalinga Institute of Medical Sciences (KIMS), KIIT University, and conducted according to the Helsinki declaration. Voluntary consent forms were signed before each collection of samples

**Silencing of TRF2 and p38 inhibition in HNSCC cells**

TRF2 silencing was done in HNSCC cell lines as per manufacturer’s instructions (Dharmacon) and laboratory established ^1^. For TRF2 silencing, cells to be transfected were seeded at a density of 1 X 10^4^ cells per well. Transfection was done with DharmaFECT transcription reagent (Dharmacon). Scrambled siRNA (siGENOME, Dharmacon) and TRF2 siRNA (siGENOME Human TERF2, Dharmacon) were used for silencing. The transfection time was 48 hours. The effect of silencing was evaluated by western blot, Quantitative Real Time PCR and immunofluorescence assay.

**Table listing the siRNA used and the target sequences**

| **siRNA** | **Target Sequences** |
| --- | --- |
| siGENOME Human TERF2 | GAAGUGGACUGUAGAAGAA |
|  | GGAAGCUGCUGUCAUUAUU |
|  | GGAUCAGCUAUACAAUGUG |
|  | GAAGACAGUACAACCAAUA |
| SiGENOME Non-Targeting siRNA | UAGCGACUAAACACAUCAA |

For p38 inhibition, SCC-131 cells were seeded at a density of 1 X 10^4^ cells per well. Cells were treated with 10µM of p38 inhibitor (SB 203580) for 4 hours after which the treatment media was removed and cells were used for respective experiments. The effect of silencing was evaluated by Western blot, Quantitative Real Time PCR and immunofluorescence assay.

**Protein Extraction /SDS-PAGE/Western Blot**

To check the expressions of proteins, western blot analysis was carried out for patient tissues and cell line as described earlier ^2^. Protein lysate was prepared from cells and tissue samples. The pellet was resuspended in RIPA lysis buffer. 50 μg of protein lysates were separated on 12% SDS-PAGE and transferred onto a PVDF membrane. The membrane was blocked with 5% skimmed milk in PBST for 1hr at room temperature (RT) and then probed with the primary antibody (1:2000) for 4hr at RT or at 4°C overnight. The membrane was washed with PBST and probed with secondary antibody (1:4000) for 2hrs at RT. The blots were visualized by enhanced chemiluminescence using X-ray film (Kodak, India).

**Immunocytochemistry**

Immunocytochemistry assay was performed as described earlier ^3^. Cells were grown on glass coverslips to 60%-80% confluency. The media was decanted and the cells grown on the coverslips were washed with 1X PBS for three times. Cells were then fixed with 1:1 acetone: methanol for 15 minutes at -20^◦^C followed by 1X PBS wash. Cells were permeabilized with 0.25% PBST for 10 minutes at RT followed by 1X PBS wash. After permeabilisation cells were blocked with 5% BSA (Bovine Serum Albumin) for 30 minutes. Cells on the coverslip were then probed with the primary antibody (1:2000) at 4°C overnight. Cells were washed with PBS and probed with FITC/TRITC fluorophore tagged secondary antibodies (1:4000) for 1hr at RT. Coverslips were counterstained with 4, 6-diamidino-2-phenylindole (DAPI), mounted, sealed and viewed under fluorescence microscope Olympus (BX 61) and images were captured using Image Pro Express software. The cell fluorescence was measured using ImageJ software. Total Corrected Cell Fluorescence (CTCF) was calculated using the formula

CTCF = Integrated Density – (Area of selected cell X Mean fluorescence of background readings) ^4^.

**RNA extraction and Quantitative Real Time PCR**

RNA extraction and quantitative real time PCR was done as described earlier ^2^. Total RNA was extracted from the frozen stored tissues and HNSCC cells with TRIsure reagent (BIOLINE) in accordance with the manufacturer’s instructions. Reverse transcription was performed in total volume of 20µl using 2µg of total RNA by TETRO cDNA synthesis kit (BIOLINE). Real time PCR was carried out on Step-One Plus (ABI) for p38 alpha and TRF2 using KAPA SYBR^®^  FAST qPCR Kit Master Mix (2x) Universal (KapaBiosysytems). β-actin was used as the housekeeping gene PCR conditions were set as per manufacturer’s instruction. Quantification was performed using the 2^-ΔΔCT^ method.

| **Genes** | **Forward Primer** | **Reverse Primer** |
| --- | --- | --- |
| p38α | 5’-GTGCCCGAGCGTTACCAGACC-3’ | 5’- CTGTAAGCTTCTGACATTTC-3’ |
| TRF2 | 5’- GTACCCAAAGGCAAGTGGAA-3’ | 5’- TGACCCACTCGCTTTCTTCT-3’ |
| β-actin | 5’-TCACCCACACTGTGCCCATCTACGA-3’ | 5’-CAGCGGAACCGCTCATTGCCAATGG-3’ |

**Co-Immunoprecipitation (Co-IP)**

The interactions between p-p38 and TRF2 were measured in the protein lysate of SCC-131 Head and Neck cancer cells and patient tumor tissue. Protein lysate from cells as well as tissues were incubated with with different antibodies as indicated overnight at 4^0^C. The blocked protein G agarose beads were incubated with IgG rabbit (for control) and IgG mouse (for control) for 4 hours at 4^0^C. The IgG derived from the same species as the primary antibody was used as the negative control. The protein-antibody solution was incubated with the blocked protein G agarose beads for 4 hours at 4^0^C. Beads were washed thrice with wash buffer. Beads were then suspended in a SDS-sample loading buffer and the soluble proteins were separated by 12% SDS-PAGE, transferred onto a PVDF membrane, and probed with an anti-p-p38, anti-p38 and anti-TRF2 antibody.

**Cell Viability Assay**

Cells were seeded at a density of 8000 cells/well. At 60-70% confluency cells were treated with increasing concentrations of cisplatin for 24 hours. After 24 hours of treatment, 0.5 mg/ml of MTT (3-(4, 5-Dimethylthiazol-2-yl)-2, 5-Diphenyltetrazolium Bromide) was added to each well and was incubated for 3-4 hours for the formation of formazon crystals. The crystals were dissolved with dissolution solution (11gm SDS dissolved in 50 ml isopropanaol and 50ml 0.02M HCl). The absorbance was measured at 570 nm in ELISA reader (Biotek,Germany). Experiments were performed in triplicate. IC_50_ values were determined by using GraphPad Prism 6 software.

**Clonogenic survival assay**

Colony formation capacities of cells were determined by using clonogenic survival assay. Briefly, 500 cells/well were seeded in a 6-well cell culture plate and allowed to adhere overnight at 37ºC. The cells were then treated with increasing concentrations of cisplatin (1-15 µM) for 24 hours. After that, media containing drug was replaced with fresh media and cells were allowed to form colonies for 7-8 days. Thereafter media was removed and stained with 0.2% crystal violet prepared in methanol. Then, the wells were washed with distilled water, and colonies were counted using gel documentation system (UVP, Germany). Experiments were performed in triplicates, and the data were represented as number of colonies formed per 500 cells and percent survival relative to control.

**Dead live staining**

Cells were seeded at a density of 1× 10^5^ cells per well. Cells were treated at 60-70% confluency with increasing concentration of cisplatin for 24 hours. After 24 hours cells were harvested and resuspended in 1X PBS. A positive control was prepared by fixing a population of cells using 70% ethanol followed by incubation at 4^0^C for 90 minutes. Half of the fixed cells were stained with 1ug/ml Propidium Iodide (50ug/ml stock) for 20 min in dark at room temperature and half of the cells were unstained. 1ug/ml Propidium Iodide (PI) was subsequently added to all the samples and was kept in dark for 20 minutes. Cells were then washed with PBS and Flow cytometric analysis was performed using FACS CANTO II (Becton & Dickinson, CA, USA). Analysis of data was done by FACS diva software.

**Cell cycle and apoptotic analysis**

Cells were seeded at a density of 1× 10^5^ cells per well. Cells were treated at 60-70% confluency with increasing concentration of cisplatin for 24 hours. After 24 hours cells were harvested and fixed with ice chilled 70% ethanol and then incubated for 30 minutes at −20 °C. Then, cells were washed with 1X PBS and stained with 0.1 mL of 50 μg/mL Propidium Iodide (PI) containing 0.05% RNase and then incubated in dark at 37 °C for 30 minutes. Cells were sorted by FACS (FACS CANTO II, Becton & Dickinson, CA, USA). Analysis of data was done by FACS diva software.

**Protein sequence Retrieval**

The information regarding the p38 (Q16539) and TRF2 (Q15554) were publicly available at UniProt database and NCBI. The protein sequences for human p38 (Mitogen-activated protein kinase 14-MAPK14) and TRF2 (Telomeric repeat-binding factor 2-TERF2) were retrieved from ftp server at National Centre for Biotechnology Information (NCBI) in the form of FASTA format. To find out the domain-domain interactions, the protein sequences are subjected for Blastp search using default parameters (http://blast.ncbi.nlm.nih.gov/Blast)^5^. The conserved domains exhibited similar functions in p38 and TRF2 proteins were further used for docking studies. The PDB structures of p38 and TRF2 were also downloaded from Protein Data Bank (http://www.rcsb.org/) for further analysis.

**Homology modeling to obtain 3D structure of p38 and TRF2 protein**

To predict the 3-dimensional structure of p38 and TRF2, PDB structures of p38 (PDB ID: 1BL6) and TRF2 (PDB ID: 1VF9) were submitted to Phyre2 server for homology modelling (http://www.sbg.bio.ic.ac.uk/phyre2) ^6^. It identifies and predicts the 3-dimensional structure of protein sequences employing HMM–HMM alignment techniques. For the tertiary structure prediction, we chose the normal mode of Phyre2 for generating the 3-dimensional model of our proteins. PyMOL molecular graphics program (https://www.pymol.org) was used for structure visualization, assessing the complex interactions and measuring the distances between interacting amino acid residues ^7^.

**Localization of energetic frustration in protein molecules**

To evaluate the degree of local frustrations illustrated by spatial local interactions in the two proteins, namely Mitogen-activated protein kinases ‘p38’ (PDB Id: 1BL6) and Telomeric repeat-binding factor 2 ‘TRF2’ (PDB Id: 1VF9), the Frustratometer web server (http://www.frustratometer.tk) has been used ^8^.The Protein Frustratometer is a tool that helps to localize energetic frustration in protein molecules. Moreover, it is an energy landscape theory inspired algorithm that directed to quantify the sites of high local frustration, minimal local frustration and neutral contacts indicated in protein molecules. The sites containing the highest local frustration designated as biologically important regions that were involved in binding or allostery. In contrast, minimally frustrated linkages comprised a stable folding core of the protein molecule which was noticed as highly conserved in their conformation.

**Protein-protein interaction through molecular docking**

Molecular docking between p38 and TRF2 were performed using ClusPro 2.0 protein-protein docking server (https://cluspro.bu.edu) with default parameters ^9^. The server utilizes the structural files or PDB IDs of query interacting partners as input and performs rigid body docking to give a docked model. Twenty five conformations were obtained by using TRF2 (PDB ID: 1VF9) as receptor and p38 (PDB ID: 1BL6) as ligand. The best conformation was selected and further utilized for generating molecular interactions plot between P38 and TRF2 using Dimplot program of LIGPLOT software ^10^ using default parameters.

To assess the stability, binding interactions among the amino acid residues found at active and inactive sites of p38 MAPK with TRF2 and their interaction may result in any conformational change, similar molecular docking approach was used. The unphosphorylated (PDB ID: 1BL6) and phosphorylated (PDB ID: 3PY3) structures of p38 were docked with TRF2 (PDB ID: 1VF9) and Dimplot program was used for plotting the hydrogen bonds, ionic and other interactions between binding interfaces.

**References**

1 Saha A *et al*. Role of TRF2 in efficient DNA repair, spheroid formation and Cancer Stem Cell maintenance. *Oncomedicine* 2017; **2**: 71–79.

2 Padhi S *et al.* Clinico-Pathological Correlation of β-Catenin and Telomere Dysfunction in Head and Neck Squamous Cell Carcinoma Patients. *J Cancer* 2015; **6**: 192–202.

3 Saha A, Shree Padhi S, Roy S, Banerjee B. HCT116 colonospheres shows elevated expression of hTERT and β-catenin protein - a short report. *J Stem Cells* 2014; **9**: 243–51.

4 Measuring cell fluorescence using ImageJ — The Open Lab Book v1.0. http://theolb.readthedocs.io/en/latest/imaging/measuring-cell-fluorescence-using-imagej.html (accessed 24 Jan2018).

5 Altschul SF *et al.* Gapped BLAST and PSI-BLAST: a new generation of protein database search programs. *Nucleic Acids Res* 1997; **25**: 3389–402.

6 Kelley LA, Mezulis S, Yates CM, Wass MN, Sternberg MJE. The Phyre2 web portal for protein modeling, prediction and analysis. *Nat Protoc* 2015; **10**: 845–858.

7 Delano WL. PyMOL: An Open-Source Molecular Graphics Tool. http://www.ccp4.ac.uk/newsletters/newsletter40/11_pymol.pdf (accessed 8 May2017).

8 Jenik M *et al*. Protein frustratometer: a tool to localize energetic frustration in protein molecules. *Nucleic Acids Res* 2012; **40**: W348–51.

9 Comeau SR, Gatchell DW, Vajda S, Camacho CJ. ClusPro: a fully automated algorithm for protein-protein docking. *Nucleic Acids Res* 2004; **32**: W96–9.

10 Wallace AC, Laskowski RA, Thornton JM. LIGPLOT: a program to generate schematic diagrams of protein-ligand interactions. *Protein Eng* 1995; **8**: 127–34.
